# Better self-concept, better future choices? Behavioral and neural changes after a naturalistic self-concept training program for adolescents

**DOI:** 10.3758/s13415-021-00946-1

**Published:** 2021-09-27

**Authors:** L. P. E. Van der Aar, S. Peters, A. I. Becht, E. A. Crone

**Affiliations:** 1 Department of Developmental Psychology, Leiden University, Leiden, the Netherlands; 2 Institute of Psychology, Brain and Development Research Center, Leiden University, Wassenaarseweg 52, 2333 AK Leiden, The Netherlands; 3 Erasmus School of Social and Behavioural Sciences, Erasmus University Rotterdam, Rotterdam, the Netherlands; 4 Research Center Adolescent Development, Utrecht University, Utrecht, the Netherlands

**Keywords:** Adolescence, Self-concept training, Gap year, Educational decision-making

## Abstract

A large number of adolescents experience difficulty when choosing a suitable higher education program that matches their self-views. Stimulating self-concept development could help adolescents to increase their chances of finding a suitable major. We addressed this issue by examining the effects of a naturalistic self-concept training within a gap year context on behavioral and neural correlates of self-evaluations, as well as the long-term effects for future educational decision-making. In total, 38 adolescents/young adults (ages 16-24 years) participated in a 4-wave longitudinal study, with lab visits before, during, and after the training, including behavioral assessments and fMRI. During fMRI-scanning, they rated themselves on positive and negative traits in academic, (pro)social, and physical domains, and additionally filled out questionnaires related to self-esteem and self-concept clarity. Results showed that the positivity of domain-specific self-evaluations, self-esteem, and self-concept clarity increased during the training. Second, participants with lower medial PFC activity during self-evaluation before training showed larger self-esteem increases over the year. Moreover, mPFC activity increased after training for the evaluation of positive but not negative traits. Furthermore, individual differences in the rate of change (slope) in self-concept clarity and social self-evaluations positively predicted social adjustment to college and academic performance 6 months after training. Together, these findings suggest that self-concept can be modulated in late adolescents, with an important role of the medial PFC in relation to enhanced positive self-evaluations, and self-concept clarity as a predictor of future educational outcomes.

**Supplementary Information:**

The online version contains supplementary material available at 10.3758/s13415-021-00946-1.

## Introduction

Adolescence is a period in life during which the ability for self-reflection is still developing (Sebastian et al., 2008). How adolescents view and evaluate themselves can play an important role in various life outcomes. For example, many studies have demonstrated positive relations between the positivity of self-evaluations and general well-being, mental health, as well as motivation and achievement in school (Marsh & Martin, 2011; Orth et al., 2012). Within this school domain, the transition into higher education is an important life change that almost all adolescents have to face but that remains relatively understudied. In this process, having a clear and coherent self-concept appears crucial in order to choose a suitable future educational or career path (Eccles, 2009), referred to as educational decision-making in this study. For example, in a recent study, we showed that the overall evaluation of the self (self-esteem) and the clarity of self-beliefs(self-concept clarity) were significantly lower in adolescents who experienced difficulties with educational decision-making compared with peers who already successfully transitioned into higher education (van der Aar, Crone, & Peters, 2019a)

The fact that educational decision-making can be challenging, especially at a relatively young age, also is reflected in an increasing number of adolescents who are postponing this choice by taking one or multiple gap years before starting a major in higher education. In the Netherlands, statistics show that one in ten students had taken a gap year before starting college education in 2017, an increase of almost 6% over 10 years (Researchned, 2018a). The majority of adolescents who took a gap year indicated the main reason for their gap year was to gain more time to reflect upon themselves and their options in order to make a suitable choice for their future (Researchned, 2018a). This raises the question whether a gap year also could be used as a targeted intervention period explicitly focused on self-concept development, thereby increasing adolescents’ chances of finding a suitable major. Therefore, the goal of this study was to examine the effects of a self-concept training within a gap year context to prepare for future educational decision-making.

### A neuroscientific approach to self-concept and self-concept training

Adolescence is an especially interesting period to investigate self-concept development. Decades of research have shown that this phase in life is particularly important for identity development and a time where the structure and evaluation of the self are still highly changeable (Becht et al., 2016; von Soest et al., 2016). Our understanding of adolescence as an inflection period has benefited from research on brain development, which demonstrated that the cortical midline regions of the brain, spanning from anterior cingulate cortex (ACC) to posterior cingulate cortex (PCC), but specifically the medial prefrontal cortex (mPFC), play an important role in self-evaluation(Pfeifer & Berkman, 2018; Romund et al., 2017; van der Cruijsen et al., 2018). Prior studies showed that activity in these regions continue to develop during adolescence (Pfeifer et al., 2013; Pfeifer & Berkman, 2018) consistent with research showing that self-concept has a prolonged developmental trajectory (Van Doeselaar et al., 2018).

Recent studies have shown dissociable brain activity in regions related to a specific self-concept domain (e.g., self-evaluations in academic, social, or physical appearance domains) or valence of traits (e.g., positive versus negative self-evaluations). For example, evaluating academic traits, such as “I am smart” was shown to elicit specific activity in the PCC/precuneus(van der Aar, Peters, et al., 2019b; van der Cruijsen et al., 2018). Additionally, more specific parts of the mPFC have been linked to differences in valence and self-relevance of traits, such that stronger activation in the ventral part of the mPFC (vmPFC) has been related to more positive as well as more self-relevant self-descriptions (D’Argembeau, 2013; van der Cruijsen et al., 2018).

Furthermore, prior studies showed that self-concept can be dissociated in *direct* (how do I think about me?) and *reflected*self-concept (how do I think that others think about me?) (Jankowski et al., 2014; Pfeifer et al., 2009). Especially the temporal-parietal junction (TPJ), a region of the social brain network that is involved in perspective taking (Schurz et al., 2014), plays an important role in both direct and reflected self-evaluations, in interaction with behavioral positivity ratings. For example, van der Cruijsen et al. (2019) showed that the TPJ was more strongly activated for reflected than direct self-evaluations when adolescents were less positive about themselves. Possibly, these results indicate that these adolescents are more concerned about the opinions of others compared to adolescents who are more positive about themselves.

Taken together, the neural processes underlying self-evaluation appear specifically targeted in the mPFC for positively valenced self-evaluations(Van der Cruijsen et al., 2018), PCC/precuneus for academic self-evaluations(van der Aar et al., 2019a, b) and TPJ for reflected self-evaluations(Jankowski et al., 2014; Van der Cruijsen et al., 2019), but these regions form part of a larger network with strong interconnections (Sebastian et al., 2008). It is not yet understood how these regions are sensitive to changes in (domain-specific) self-evaluations, self-esteem and self-concept clarity over time. It was previously suggested that the developing brain is influenced by cognitive and social experiences throughout adolescence, with considerable implications for treatment and intervention (Jolles & Crone, 2012). However, the transitional phase of late adolescence into young adulthood is relatively understudied, especially in brain research (Veroude et al., 2013). Consequently, we currently have little understanding of whether in late adolescents, self-concept can be fostered through training and which underlying neural mechanisms would drive these changes. Therefore, in this study we examined both the neural and behavioral effects of self-concept training in a gap year context.

### Gap year and self-concept interventions

To our knowledge, no studies have investigated the effects of a transitional gap year that consists of a self-concept training. Existing research on self-concept training has mostly been performed within school contexts, targeting school-age populations (up to 18 years). Review studies have concluded that these intervention programs are generally successful in enhancing general self-esteem and domain-specific aspects of self-concept, such as improving self-perceptions within the academic domain (Haney & Durlak, 1998; O’Mara et al., 2006). Harter (2012a, 2012b), and Bos et al. (2006) suggested that important working mechanisms for self-concept interventions are related to the *cognitive* and *social* determinants of self-concept. That is, they argue that self-concept interventions should be aimed at changing both cognitive aspects (e.g., reframing dysfunctional self-beliefs), and social factors (e.g., increase of social support, internalization of positive opinions of others) to have a significant positive outcome.

These interventions, however, are rarely only focused on changing self-concept but often are imbedded in larger intervention programs aimed at promoting social-emotional skills in young people. An example of a popular, worldwide used group of programs is SEL (Social Emotional Learning; Durlak et al., 2011). SEL programs are school-based and aim to foster competencies related to the self (e.g., self-awareness and self-management, decision-making skills) and others (empathy, perspective-taking, relationship skills). These competencies, in turn, are expected to improve academic performance, adjustment, self-perceptions, and positive social behaviors (Gutman & Schoon, 2013). Several meta-analyses have shown generally positive findings, with significantly improved social and emotional skills, increased self-confidence, and academic performance compared with control participants (Durlak et al., 2010; Durlak et al., 2011).

### Current study

The current study made use of an ecologically valid existing gap-year program in the Netherlands, called “The Gap Year program” (www.breekjaar.nl). This program provides a 10-month training program for adolescents aged 16–24 years who experience difficulties with making future academic and career choices. It is based on the concept of “folk high schools” found in Scandinavian countries, which promote lifelong learning: the idea that schools should educate for life. The Gap Year program has a large overlap with SEL programs; it focuses on fostering self-concept development within the larger context of training social competences (for more information on the content of the program, see method section: training program).

For this preregistered study (see https://osf.io/8mspn/), we examined changes in self-concept in terms of domain-specific self-evaluations (academic, physical, prosocial, and social), as well as a more global evaluation of the self (self-esteem), and changes in the structure of the self (self-concept clarity). The main objectives were to (1) test whether self-concept training, as observed in a naturalistic setting within a gap year context, would be beneficial for the development of domain-specific self-evaluations, self-esteem and self-concept clarity, (2) examine the neural circuitry associated with self-processing before and after the training, and to test whether changes in activity in medial PFC, precuneus and TPJ were correlated with changes in the positivity of domain-specific self-evaluations and self-esteem, and (3) test the predictive value of changes in behavioral indices of self-concept for future educational decision-making.

**Fig. 1:**
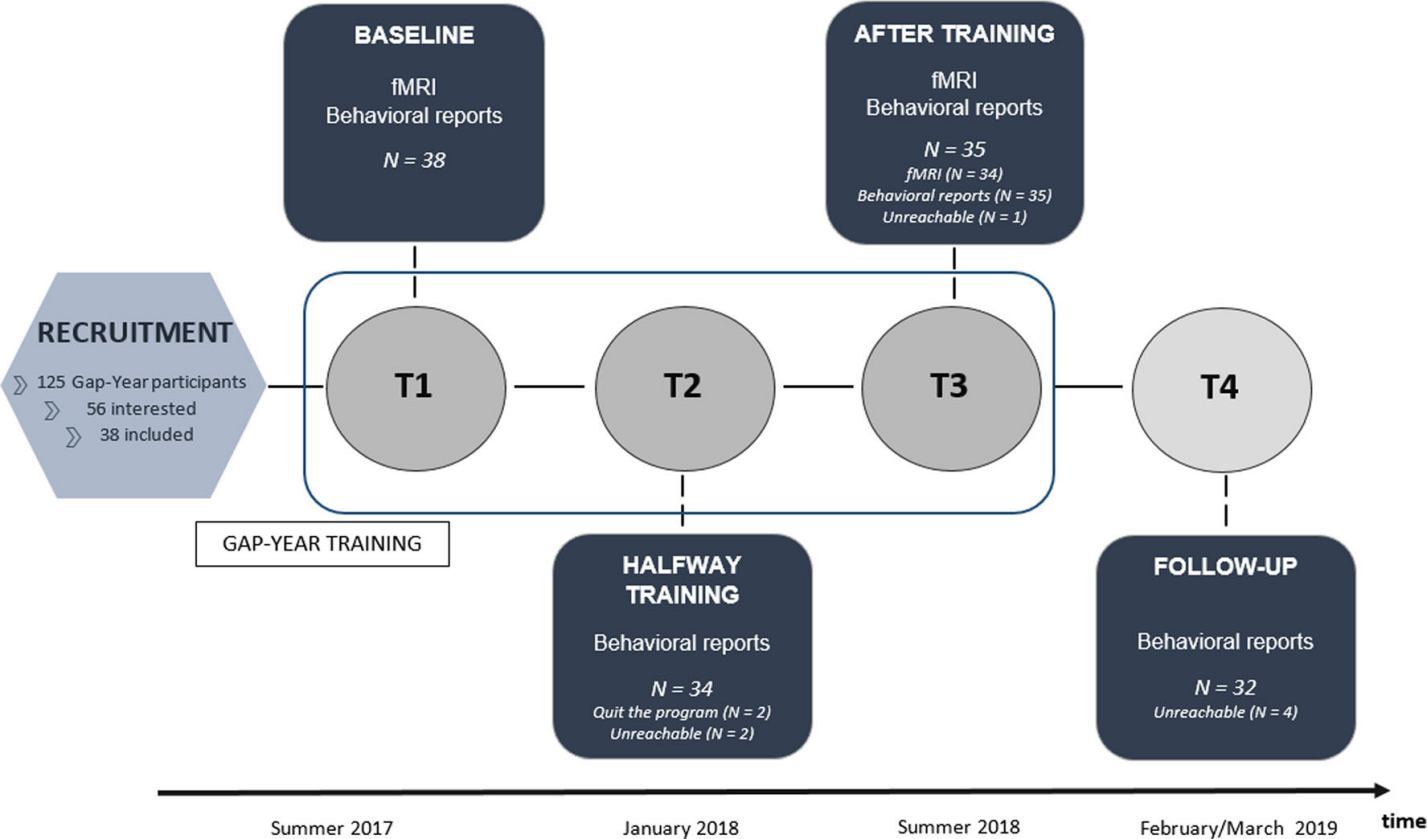
Study design with time-line and overview of participants included at each wave

To test for training effects, we examined the behavioral and neural correlates of self-concept before the start of the training program (baseline; time point 1 (T1)), and after the program (10 months; T3). For this purpose, participants completed a task that included evaluations of direct and reflected self traits across academic, (pro)social and physical trait domains (based on van der Cruijsen et al., 2018) during fMRI scanning. Furthermore, we additionally collected behavioral data halfway through the program (5 months; T2) and at follow-up (18 months; T4) to follow the time course of changes, and the predictive value for the final time point (see Fig. 1 for a visualization of the study design).

#### Preregistered hypotheses

Our preregistered hypotheses were as follows: behaviorally (aim 1), we expected that that the focus the self during the training would result in a more positive self-concept after training (Bos et al., 2006), reflected in an increase in the positivity of all domain-specific self-evaluations (from both direct and reflective perspectives), self-esteem, and enhanced self-concept clarity after training. For all variables, we expected linear increases across the training year. As the training takes place in a group, we expected that participants would benefit from the advantages of group counseling, such as peer acceptance and increased social skills (Forsyth, 2015; Hoag & Burlingame, 1997). Therefore, we expected that the increase in the positivity of self-evaluations would be most significant for the social domain.

In terms of neural activity (aim 2), we focused on changes in the medial PFC, precuneus, and TPJ as three regions of interest that were previously shown to play an important role in self-evaluation(Pfeifer & Peake, 2012). First, we predicted that thinking about self (versus a control task) would be associated with increased activity in medial PFC. We previously demonstrated based on the data from the first time point (before training) that medial PFC activity was positively correlated with self-esteem ratings (van der Aar, Crone, & Peters, 2019a). Therefore, we predicted that increases in self-esteem would be associated with increases in medial PFC activity during self-evaluation after training. Additionally, based on literature relating self-relevance of traits to increased (ventral) medial PFC activity, we expected that that more positive self-evaluations would be reflected in increased mPFC activation for evaluating positive versus negative self-traits after training (D’Argembeau, 2013). Second, we predicted that precuneus would show increased activity for evaluating academic traits specifically (versus a control task), and would be correlated with changes in behavioral positivity of self-evaluations in the academic domain (van der Aar et al., 2019b). Third, as a reflection of the internalization of positive opinions of others, we expected increases in right TPJ activity for direct versus reflected self-evaluations that would be associated with increases in behavioral positivity of self-evaluations( van der Cruijsen et al., 2019).

Finally, with regard to the predictive value of changes in self-concept for successful educational decision-making (aim 3), we expected that individual differences in changes in self-esteem and self-concept clarity during the year of training (T1, T2, T3) would be predictive of outcomes related to educational decision-making on T4. That is, we expected participants with higher starting levels of self-esteem/self-concept clarity and/or stronger increases in self-esteem/self-concept clarity levels to show more positive outcomes related to *general* outcomes (satisfaction with the chosen study or career path, and satisfaction with life) as well as more positive *academic* outcomes (related to study commitment, academic motivation, adjustment, engagement, and performance). For more information on the outcome measures, see method section. Additionally, we focused specifically on the predictive value of the social domain and academic domain for positive outcomes, because of the embedding of the program in social competence training and the focus on academic outcomes. For the social domain, we expected that participants with higher starting points and/or stronger increases in positivity scores would show increased life satisfaction as well as better social adjustment to college (Proctor et al., 2009). For the academic domain, we expected participants with higher starting points and/or stronger increases in positivity scores to show better academic motivation, academic adjustment to college and academic performance (Huang, 2011; Valentine et al., 2004; Wouters et al., 2011).

## Method

### Participants

A total of 38 late adolescents/young adults in the age range of 16–24 years (*M*_*age*_ = 18.73; SD = 1.47; 24 females) participated in this 4-wave longitudinal study. Results from the first data wave have been published previously (van der Aar et al., 2019a). Participants were recruited in collaboration with Foundation Gap Year before they started their gap year training program in September 2017. For recruitment, we were dependent upon the number of places the Gap Year program has available per year and the application period for the program. For the year 2017-2018, the program had a capacity of 125 spaces. Adolescents were asked at their intake conversation with the program whether they were interested in participating in this study. If they showed interest, their contact information was sent to us. We started recruitment in June and continued recruitment and inclusion of participants until the program started at the beginning of September.

In total, 56 adolescents showed interest in our study (45% of the total number of applications). Of these 56, 38 adolescents were ultimately included in the study. Reasons for exclusion were MRI contraindications (*N* = 8) or current use of psychotropic medications *(N* = 2). Some adolescents withdrew (*N* = 3) or could not be reached before the start of the program (*N* = 5). We chose to include individuals with a clinical diagnosis (*N* = 7) as long as they were not on medication at the time of testing, because studies have shown that experiencing problems with educational decision making or career indecision often is confounded with other psychological problems (Gati et al., 2011; Scholtens et al., 2013). Diagnoses included ADHD (*N* = 2), ADD (*N* = 3), AD (*N* = 1), and depression (*N* = 1). We included right-handed (*N* = 33) as well as left-handed individuals (*N* = 5) with the criterion that they were able to use the button box with their right hand.

All 38 participants graduated from high school. Fifteen participants reported that they started at least one college major but dropped out. Twenty-three participants took part in the program directly after high school. They all participated in an MRI session before the start of the program (T1). Behavioral data at T2 were collected from 34 participants (2 dropped out of the program; 2 were unreachable). At the end of their gap year, all 36 remaining participants were invited for a second MRI session at T3. MRI data were collected from 34 participants; 1 participant only filled out questionnaires due to MRI contraindications. At T4, questionnaire data were collected from 32 participants (4 were unreachable). See Fig. 1 for an overview of the inclusion numbers at each wave. Additionally, more information about the demographics and psychological status of the total sample, the drop-outs, and the subgroups (started their gap year directly after high school or tried college majors before) at T1 can be found in Table S.1.

At T1, an estimation of IQ was obtained based on two subtests of the Wechsler Adult Intelligence Scale-III (Similarities and Block Design). Estimated IQ scores were in the normal range (*M*_*IQ =*_ 104.47; *SD*_*IQ*_ = 9.5; range = 85–127.50). At each time point, written, informed consents were provided by the participants themselves or by both parents for minors. Participants were screened for MRI contraindications, had normal (or corrected to normal) vision, were fluent in Dutch, had no neurological impairments, and were not taking psychotropic medication. The study was approved by the University Medical Ethics Committee.

### Training program

The Gap Year program is a Dutch nonprofit organization that provides training programs for adolescents who have dropped out of higher education and experience difficulties with making future academic and career choices. Their goal is to help adolescents gain confidence and more self-knowledge and to guide them toward making a well-suited future academic or career choice. They have locations in multiple cities in the Netherlands (Amsterdam, Utrecht, and Eindhoven) and can place around 120 participants per year. Participants of this program follow a 10-month training (September–June) focused on personal development and start working on improving their self-esteem and decision-making abilities.

The training consists of multiple projects that are scheduled across the year, each with a focus on the self, as well as a travel period. Examples of these projects are “Project me,” where adolescents learn more about themselves (their traits, talents, goals) together with a coach; “Project me and the other,” where it is explored how participants relate to others (e.g., peers or society); “Project me and the world,” where participants are challenged to come out of their comfort zone and learn more about themselves and their behaviors while exploring the world in a 6-week travel period; and “Project me and the future,” which focuses on the process of decision-making with an emphasis on choosing a future study or career path. The training takes place 3 days per week in groups with a maximum of 30 adolescents. Each group is mentored by three coaches. In addition, participants can get help from a study advisor and the coaches for individual sessions.

### fMRI Task

Self-processing was studied with an fMRI task in which short sentences were presented that described positively or negatively valenced traits or competencies in four specific domains: academic (e.g., “I am smart” or “I find school difficult”), physical (e.g., “I am attractive” or “I am overweight”), prosocial (e.g., “I like to help others” or “I ignore other people’s problems”), social (e.g., “I am spontaneous” or “I feel lonely”), and one global domain (e.g., “I am happy with myself” or “I am insecure”). Each domain contained 20 traits (10 positive and 10 negative), making a total of 100 trait sentences. This task is part of the Leiden Self-Concept Study where the academic, physical, and prosocial domain have already been used (for more information and validation of the traits in these domains see van der Cruijsen et al., 2018). For the current study, the social and global domain were added to obtain a more complete representation of the development of self-evaluation in domains that are expected to be significant during the gap year training. However, as the content of the global domain had a large overlap with our measure of self-esteem (correlations between 0.81 and 0.88 at each time point), we decided to omit the global domain and only focus on the four specific domains.

The additional social domain showed good reliability measured with Cronbach`s alpha at each time point (positive valence: α = 0.89 (T1), α = 0.85 (T2), and α = 0.81 (T3); negative valence: α = 0.80 (T1), α = 0.69 (T2), α = 0.85 (T3)). For validation purposes, we correlated the social domain at T1 with a similar validated subscale of the Self Perception Profile for Adolescents (SPPA; Harter, 2012a, 2012b). Scores on the social domain correlated significantly with the subscale social competence (positive valence *r* = 0.70, *p* < 0.001.; negative valence *r* = −0.67, *p* < 0.001).

**Fig. 2:**
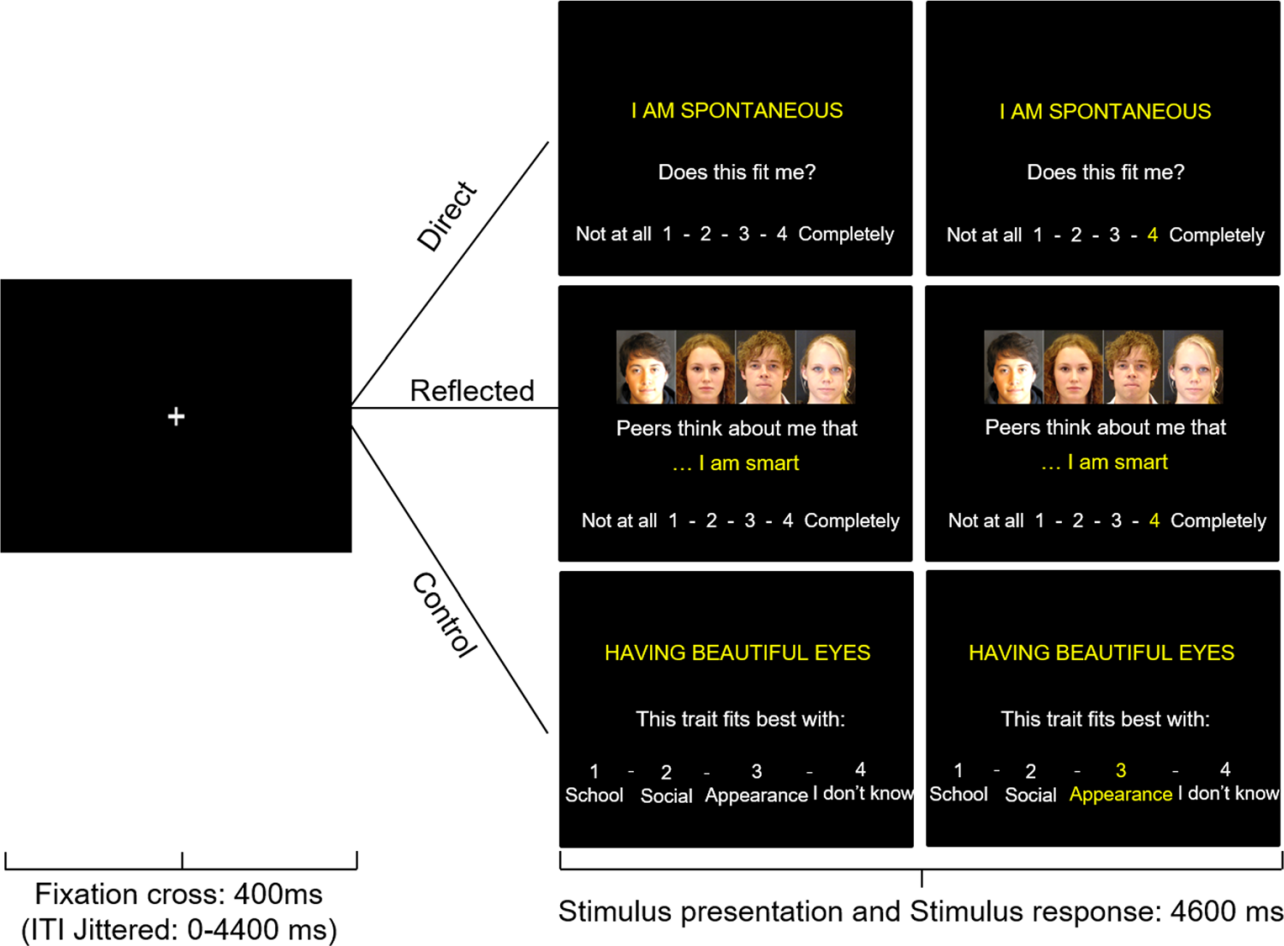
Example a trial in the Direct, Reflected, and the Control condition. Each trial started with a black screen with a jittered duration between 0 and 4,400 ms. Subsequently, a fixation cross was shown for 400 ms after which the stimulus appeared. In the Direct and Reflected conditions, participants rated on a scale of 1–4 to what extent the traits described themselves (from their own perspective or their perceived peers' perspective, respectively). In the Control condition, participants categorized the trait sentences into one of four options. The stimulus was shown for 4,600 ms. If participants responded within this timeframe, the number of their choice would turn yellow. If participants failed to respond within this timeframe, a screen with the phrase “Too Late!” was shown for an additional 1,000 ms after which the next trial would start

The task consisted of two experimental conditions (the direct self-evaluation condition, and the reflected self-evaluation condition), and a control condition (Fig. 2). In the direct self-evaluation condition, participants indicated to what extent they thought the presented trait fit them on a scale from 1 (*not at all*) to 4 (*completely*). In the reflected self-evaluation condition, participants were asked on a same scale to indicate how they thought *same-aged peers* would rate their traits. They were presented with the same trait sentences that were now preceded with the words “peers think about me that.…” Morphed pictures of unknown same-aged peers were shown during these trials to remind participants to take their peers’ perspective while evaluating their traits. In the control condition, participants were asked to categorize trait sentences instead of evaluating them. Response categories were (1) school, (2) social, (3) appearance, or (4) I don’t know. Twenty trait sentences were shown in this condition, again equally divided per valence.

The three conditions appeared in separate runs, and the order of conditions was counterbalanced across participants. The stimuli were presented in an optimized pseudorandomized order using Optseq (Dale, 1999) and were separated with a jittered black screen (0–4,400 ms). Each trial started with a 400-ms fixation cross, followed by the stimulus that was presented for 4,600 ms, consisting of the trait sentence and response options (1-4). Within this timeframe, participants could respond by pressing buttons with the index to little finger of their right hand after which the number of their choice turned from white to yellow for the remaining stimulus time. If the participant failed to respond within the 4,600 ms, they were shown the phrase “Too late!” for 1,000 ms. These trials were modeled separately and were not included in the analysis. They occurred in 0.5% of the trials in the direct condition, 0.8% of trials in the reflected condition, and on 0.2% of trials in the control condition at T1. At T3, too late responses occurred in 0.4% of direct evaluation trials, 0.5% of reflected evaluation trials, and 0.1% of control trials.

To obtain one positivity score per domain in both the direct and reflected self-evaluation conditions, scores on negative traits were reversed coded and combined with scores on the positive traits.

### Questionnaires

#### Questionnaires during training (T1, T2, T3)

##### Self-esteem

Self-esteem was assessed with a Dutch translation (Veldhuis et al., 2014) of the well-validated Rosenberg self-esteem scale (Rosenberg, 1965). This 10-item questionnaire measures global self-worth by determining both positive and negative feelings about the self. Example of items are, “On the whole I am satisfied with myself,” and “I certainly feel useless at times.” Answers were scored on a 5-point Likert scale ranging from 1 (*strongly disagree*) to 5 (*strongly agree*). After recoding the five counter-indicative items, higher scores indicated higher self-esteem. The scale had high internal consistency at each time point (Cronbach’s alpha = 0.91 at T1, 0.87 at T2, and 0.84 at T3).

##### Self-concept clarity

Self-concept clarity was measured with a Dutch translation of the Self-Concept Clarity Scale (Campbell, 1990; Crocetti et al., 2008). This 12-item questionnaire indicates the temporal stability, consistency, and clarity of someone’s self-concept. An example of an item is “My beliefs about myself often conflict with one another.” Answers were given on a 5-point Likert scale from 1 (*strongly disagree*) to 5 (*strongly agree*). Mean scores were computed such that higher scores indicate higher self-concept clarity. The scale was reliable at each time point (Cronbach’s alpha = 0.85 at T1, 0.73 at T2, and 0.87 at T3).

#### Questionnaires as outcome measures (T4)

We collected a broad range of indices related to educational decision-making to provide a global index. In order to decrease the number of tests, we submitted the variables related to *academic* outcomes to a Principal Component Analysis (PCA) to determine whether these outcome variables also could be encompassed by one or two factors (see prediction results in results section).

## General

### Satisfaction with choice

Using one question, participants were asked how satisfied they were with the study or career choice they made on a scale from 1 (*not at all*) to 5 (*very much*).

### Life satisfaction

The Satisfaction With Life Scale (SWLS; Diener et al., 1985) was used to measure global life satisfaction. The questionnaire consists of five statements concerning life satisfaction (e.g., “The conditions of my life are excellent”), which can be answered with a scale from 1 (*strongly disagree*) to 7 (*strongly agree*). Higher scores indicated higher life satisfaction. The scale showed good reliability with Cronbach`s alpha of 0.75.

## Academic

### Identity commitment

Commitment in the domain of education was measured using the commitment scale of the Utrecht-Management of Identity Commitments Scale (U-MICS; Crocetti et al., 2008). With five items, participants can indicate on a scale from 1 (*completely untrue*) to 5 (*completely true*) to what extent they feel committed to their current chosen education. An example of an item is “My education makes me feel confident about myself.” Higher scores indicate more commitment. The scale showed excellent reliability with Cronbach`s alpha of 0.93.

### Academic motivation

The Self-Regulation Questionnaire – Academic (SRQ-a; Vansteenkiste et al., 2009) was used to assess participants’ reasons for studying. The 16-item questionnaire differentiates between four types of motivation (four items per type) that can be combined into autonomous and controlled motivation behavior. For this study, we were only interested in autonomous motivation. This scale consists of identified regulation (e.g., “I am studying because it is personally important to me”) and intrinsic motivation (e.g., “I am studying because I enjoy it”). Answers could be given on a scale from 1 (*not at all important*) to 5 (*very important*). Internal consistency of the scale was good (α = 0.86).

### Student Adaptation to College

To examine to what extent participants were adjusted to their new study situation, we used a brief 20-item version (Beyers & Goossens, 2002) of the Student Adjustment to College Questionnaire (SACQ; Baker & Siryk, 1984). We focused on the scales academic adjustment (adaptation to educational demands of higher education, 10 items) and social adjustment (how well students deal with interpersonal experiences at their school environment, 10 items). Sample items are “I have been keeping up to date with my academic work” (Academic adjustment), and “I am meeting as many people and making as many friends as I would like at university” (Social adjustment). Answers could be given on a scale from 1 (*not at all*) to 5 (*very much*). Higher scores indicated better adjustment. Cronbach's alphas were 0.87 and 0.93, respectively.

### Academic Engagement

Study engagement was assessed with the shortened Dutch Utrecht Study Engagement Scale (UBES-S-9; Schaufeli et al., 2002). This 9-item questionnaire consists of three scales: vigor (“When I study, I feel full of energy”), dedication (“My study inspires me”), and 3 items to measure absorption (“Time flies when I`m studying”). Each scale comprised three items, these items were answered on a 7-point Likert-scale in a range from 1 (*Never*) to 7 (*Always*). Higher scores indicated more engagement. The internal consistency of the UBES-S-9 was excellent (α = 0.94).

### Academic Performance

An indication of academic performance was obtained with one question that asked participants about their percentage of completed courses in their first year. They could answer on a scale from 1 (*0%)* to 5 (*100%*)*.*

### Procedure

Participants were scanned two times, before (T1) and right after their gap year training (T3) with an average 10-month interval (Δ in months T1–T3: *M* = 10.4, *SD* = 0.82; Fig. 1). Before scanning, the participants were familiarized with the scanning environment with a mock scanner. They received instructions about the tasks and performed nine practice trials for each condition. Anonymity was emphasized and participants were encouraged to describe honestly how they thought about themselves.

The questionnaires used in this study were programmed in Qualtrics (www.Qualtrics.com), sent to the participants via two e-mails, and completed at home. Participants received €50 each time at T1 and T3 as compensation for the MRI scan and questionnaires. If participants could not participate in the second MRI session at T3 (e.g., because of MRI contraindications, such as braces), they could still receive €30 for filling out questionnaires. For participation at T2 and T4 (filling out questionnaires at home), participants received €30 each time.

### Behavioral training and prediction analyses

To examine changes in the positivity of domain-specific self-evaluations as well as self-esteem and self-concept clarity, we adopted a two-step procedure. First, we investigated the overall change in self-concept from the start (T1) to the end (T3) of the training year using Repeated Measures ANOVAs. Next, to get a better understanding of the developmental trajectory across the training year (T1, T2, T3), as well as individual differences in these trajectories, we conducted a series of latent growth curve models (LGM; Duncan et al., 2009) on all self-concept variables in M*plus* 8.2 (Muthén & Muthén, 2012). LGM is a highly flexible method to study longitudinal data, because it can capture both mean levels (fixed effects) of starting points (intercepts) and change (slopes), as well as individual differences around these intercept and slopes (referred to as random effects). Additionally, a benefit of LGM is that it can handle partially missing data. Concerning missing data, we conducted Little’s missing completely at random (MCAR) test on all self-concept variables, which showed a chi-square (χ^2^/df) of 0.75, indicating that it is unlikely that findings were biased as a result of missing values. Therefore, we included all participants with and without missing values in our LGM analyses and handled missing data using full information maximum likelihood (FIML).

For each self-concept variable, we examined whether linear or nonlinear, quadratic, growth curve models would best describe the data. In order to facilitate model convergence with three time-points we only estimated fixed quadratic slopes and not random quadratic slopes. We compared the different models with the AIC (Akaike Information Criterion; Akaike, 1998) and BIC (Bayesian Information Criterion; Schwarz, 1978). The models with the lowest AIC and BIC values were preferred. If the AIC and BIC were inconsistent in their support for one model, we used the sample-size adjusted BIC (ssaBIC) as an additional fit indicator to select the best fitting model. All latent growth curve models were first performed with age at T1 as a covariate of intercept and slopes to control for possible age effects. If age was insignificant, it was trimmed from the model due to reasons of parsimony.

As a second goal, we investigated whether changes in self-concept variables during the year of training (T1, T2, and T3) could predict outcome measures related to successful educational decision-making on T4. For this purpose, we saved the intercept and slope parameters of each participant from the LGMs and used these intercept and slopes as predictors of general and academic outcome measures in a set of multiple regression analyses in SPSS.

### MRI data acquisition

MRI scans were acquired on a Philips 3.0 Tesla MRI scanner with a standard whole-head coil.

Functional scans were acquired in two runs with T2*-weighted echo-planar imaging (EPI) sequence (TR = 2,200 msec, TE = 30 msec, sequential acquisition, 37 slices of 2.75 mm, FOV = 220 × 220 × 111.65 mm). The first two volumes were discarded to account for T1 saturation. After the functional scans, a high-resolution 3D T1 scan for anatomical reference was obtained (TR = shortest msec, TE = 4.6 msec, 140 slices, voxel size = 0.875 mm, FOV = 224 x 178.5 x 168 mm). Stimuli were projected on a screen behind the scanner and could be viewed through a mirror attached to the head coil. Head movement was restricted by placing foam inserts inside the coil.

### MRI data analyses

MRI data were preprocessed and analyzed with SPM8 (Wellcome Department of Cognitive Neurology, London, United Kingdom). Functional images were preprocessed using the following steps: realignment, slice-time correction, spatial normalization using segmentation parameters, and spatial smoothing with a 6-mm FWHM isotropic Gaussian kernel. The normalization algorithm used a 12-parameter affine transform with a nonlinear transformation involving cosine basis functions and resampled the volumes to 3-mm cubic voxels. Templates were based on MNI-305 stereotaxic space (Cocosco et al., 1997).

Individual participants' data were analyzed using the general linear model in SPM8. The fMRI time series were modelled as a series of zero duration events locked to stimulus onset convolved with the hemodynamic response function (HRF). Modelled events of interest for the direct self-evaluation condition were: “Academic-Positive”; “Academic-Negative”; “Physical-Positive”; “Physical-Negative”; “Prosocial-Positive”; “Prosocial-Negative”; “Social-Positive”; Social-Negative.” The same events were modelled for the reflected self-evaluation condition. For the control condition, we used one event of interest (“Control”) that was collapsed across domains and valences. Trials for which participants failed to respond in time were modelled as events of no interest. The events were used as covariates in a general linear model. In addition, we included six motion parameters as nuisance regressors. The resulting first level contrast images, computed on a subject-by-subject basis, were calculated for both time points (T1 and T3) separately and submitted to second-level group analyses. We followed all analyses steps as detailed in our pre-registration on the Open ScienceFramework: https://osf.io/8mspn/.

#### Preregistered Region of Interest (ROI) analyses

To investigate our pre-registered hypotheses regarding training effects on neural indices of self-concept, we used the Marsbar ROI toolbox (Brett et al., 2002) to create ROIs of the mPFC (x = 6, y = 59, z = 13), precuneus (x = −9, y = −52, z = 28), and right TPJ (x = 60, y = −28, z = 46). These ROIs were preregistered and based on the peaks of the clusters generated in the conjunction analysis of the contrasts direct > control and reflected > control, previously used in the larger self-concept study (Van der Cruijsen et al., 2019). This study by van der Cruijsen and colleagues used the same self-concept tasks but in an independent sample of adolescents.

We extracted the parameter estimates of these ROIs for the time points before and after gap year (T1 and T3) and investigated possible differences using repeated measures ANOVAs in SPSS. To test whether the growth trajectory of a variable during training (e.g., increasing self-esteem) would influence the change in neural activity, we added the linear slope parameter of this variable used in the LGM as a covariate of interest to the repeated measures analyses. More specifically, we added the slope parameter of self-esteem to the repeated measures analysis of mPFC activity during the contrast self > control (for both task conditions) to test the relation between mPFC activity change and levels of self-esteem. Similarly, we added the slope parameter of academic positivity to the repeated measures analysis of precuneus activity during the contrast academic > control (thinking about academic traits from both direct and reflective perspective) to test whether precuneus would show increased activity after training related to increased levels of academic positivity. Lastly, we added the slope parameter of a computed general behavioral positivity score (averaged across the positivity of self-evaluations of all four domains in both the direct and reflected task) to the repeated measures analysis of right TPJ activity during direct > reflected self-processing, to test whether behavioral increases in positivity was associated with increased right TPJ activity after training. Additionally, we explored whether training related differences in ROI mPFC and right TPJ activity differed between domains and valences. All analyses were corrected for age at T1.

#### Whole-brain analyses

In addition to our preregistered ROI analyses, we explored changes in other brain regions on a whole-brain level using flexible factorial ANOVA in SPM8. We focused on three whole-brain contrasts: one valence-based contrast (evaluating positive vs. negative traits), and two task-based contrasts (Direct > Control, and Reflected > Control). For all of these whole-brain analyses, we applied FDR cluster level correction (*p* < 0.05) at an initial uncorrected threshold of *p* < 0.001, as implemented in SPM8 (Woo et al., 2014). Results of these analyses can be found as supplementary material.

## Results

### Behavioral training results

**Fig. 3:**
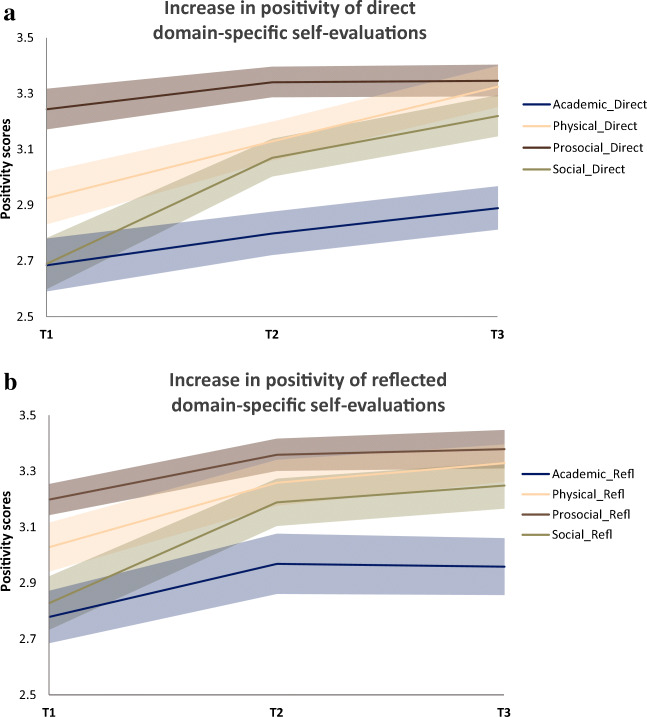
**a** Observed mean scores of the positivity of *direct*domain-specific self-evaluations on a scale from 1-4 across three time points of the gap year. LGM results showed that the positivity of physical self-evaluations increased linearly during the gap year. Social self-evaluations showed the largest increases from T1-T2. Self-evaluations of both the academic and pro-social domain remained relatively stable across the gap year. **b** Observed mean scores of the positivity of *reflected*domain-specific self-evaluations on a scale from 1-4 across three time points of the gap year. LGM results showed that the positivity of physical and prosocial self-evaluations slightly increased in the first half of the training year. The positivity of academic and social self-evaluations both showed a linear increase that levelled off toward the end of the gap year

**Fig. 4:**
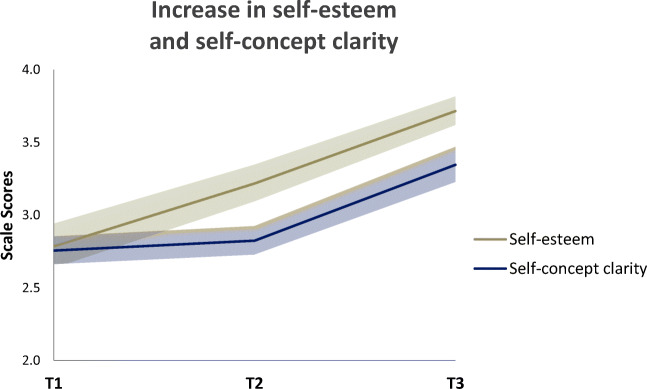
Observed mean scores of self-esteem and self-concept clarity on a scale from 1-5 across three time points of the gap year. LGM results revealed that self-esteem increased linearly, whereas self-concept clarity was relatively stable until halfway through the training (T1-T2), where after it increased

Means and standard deviations of the self-concept variables measured during training (T1, T2, T3) can be found as supplementary material Table S.2. Additionally, the observed group mean scores at each time point are illustrated in Figs. 3a, b and 4.

First, we tested for overall changes in self-concept variables before (T1) and after (T3) training for comparability with neural results. For the domain-specific self-evaluations collected during scanning, scores on negative traits were recoded and averaged with scores on the positive traits into one positivity score per domain, per task. These positivity scores were added to a 2 (Time; T1 and T3) x 2 (Task; direct and reflected) x 4 (Domain; academic, physical, prosocial, social) within-subjects Repeated Measures ANOVA. This analysis yielded a significant time x domain interaction (*F* (3, 102) = 7.96, *p* < 0.01, *η*^2^_p_ = 0.19). The time x task x domain interaction was not significant (*p* = 0.108). Post-hoc tests to unpack the time x domain interaction showed that across tasks, the positivity of self-evaluations increased significantly in all domains, although the effect size differed: academic domain (*F* (1, 34) = 10.25, *p* = 0.003, *η*^2^_p_ = 0.23), physical domain (*F* (1, 34) = 22.88, *p* < 0.001, *η*^2^_p_ = 0.40), prosocial domain (*F* (1, 34) = 5.19, *p* = 0.029, *η*^2^_p_ = 0.13) and social domain (*F* (1, 34) = 29.80, *p* < 0.001, *η*^2^_p_ = 0.47) (Fig. 3a and b).

Finally, we computed two Repeated Measures ANOVAs with Time (T1, T3) as within-subjects factor to test overall changes in the questionnaires measuring self-esteem and self-concept clarity from T1 to T3. Results showed significant increases after training in both self-esteem (*F* (1, 34) = 29.59, *p* < 0.001, *η*^2^_p_ = 0.47) and self-concept clarity (*F* (1, 34) = 13.71, *p* = 0.001, *η*^2^_p_ = 0.29) (Fig. 4).

### Behavioral longitudinal results across three time points

**Fig. 5:**
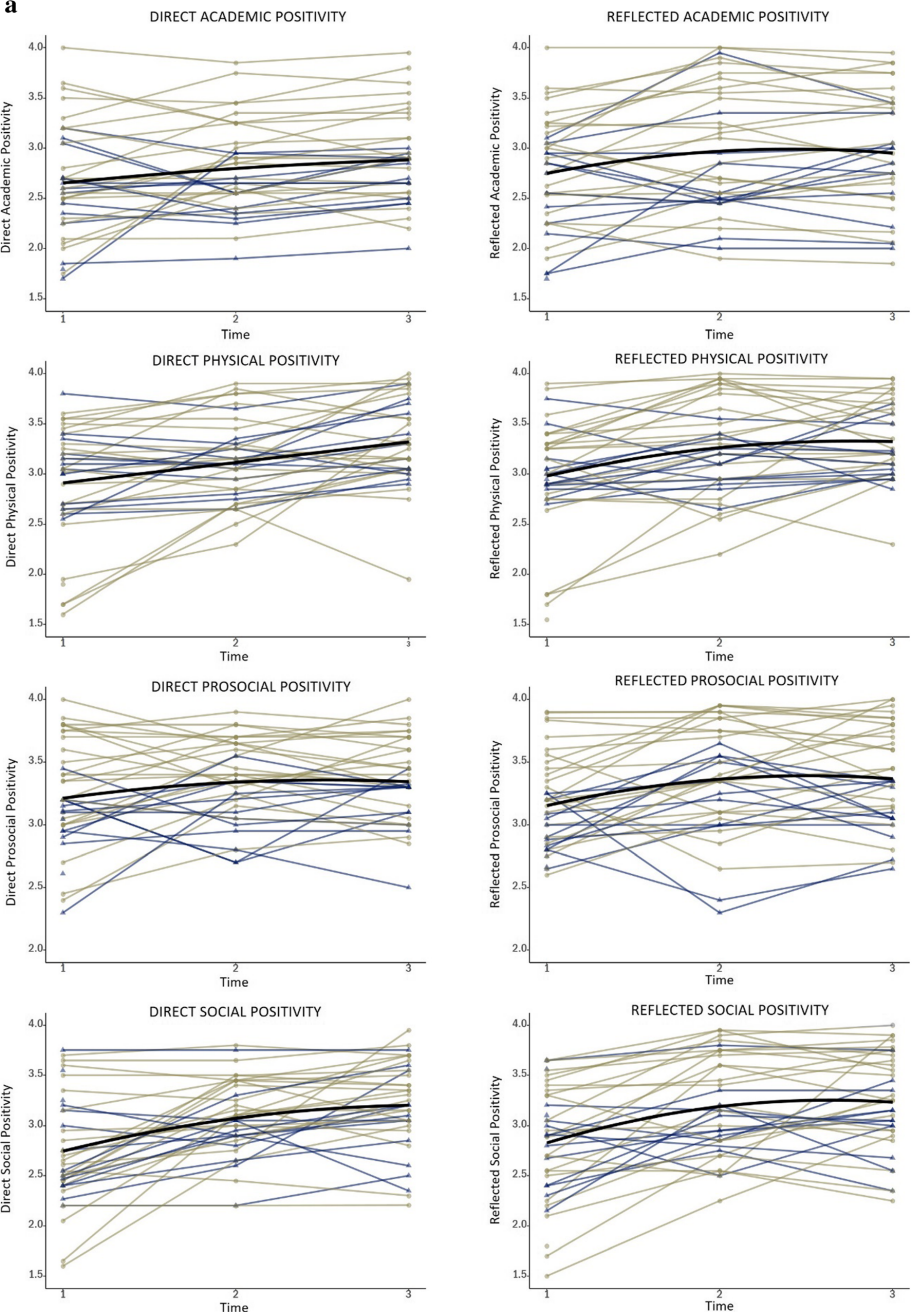

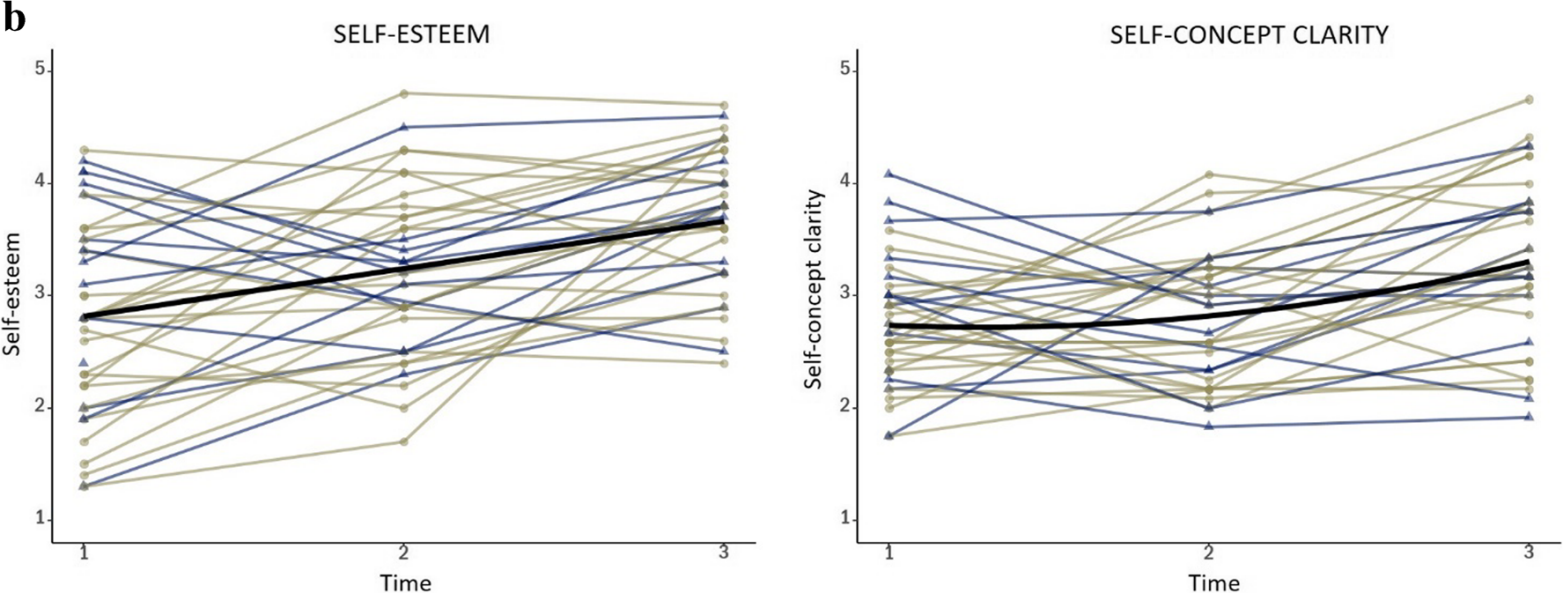
**a** Observed individual trajectories for the positivity of domain-specific direct – and reflected self-evaluations across the gap year. X-axis: time point (T1, T2, T3); Y-axis: positivity scores (1-4), yellow lines represent girls and blue lines represent boys. The black line shows the average intercept and slope. **b** Observed individual trajectories for self-esteem and self-concept clarity across the gap-year. Xaxis: time point (T1, T2, T3), Y-axis: positivity scores (1-5), yellow lines represent girls and blue lines represent boys. The black line shows the average intercept and slope

We followed up the tests of overall change in self-concept from the start (T1) to the end (T3) of the training year by examining the specific shape of growth, as well as individual differences in these growth curves, through applying latent growth curve models on all three time points (T1, T2, T3). For every self-concept variable separately, we tested whether linear or quadratic growth curve models would best describe the growth trajectory during training. For all tested models, age at T1 rendered insignificant and was therefore trimmed from the models. Table 1 shows the fit indices AIC and BIC for the different models. Table 2 shows the mean level growth parameter estimates and the individual differences in intercept and slopes. Figure 5a and b show the raw individual trajectories and the mean developmental trajectories of each variable for the entire sample.

**Table 1:** Fit indices of the latent growth curve models for all self-concept variables

Self-concept	Linear model		Quadratic model	
Variable	AIC	BIC	AIC	BIC
Direct Academic Positivity	**77.362**	**90.463**	79.200	93.938
Reflected Academic Positivity	105.137	116.600	**102.945**	**116.046**
Direct Physical Positivity	**106.228**	**119.328**	108.223	122.961
Reflected Physical Positivity	111.207	124.307	**110.543**	**125.281**^**1**^
Direct Prosocial Positivity	65.508	78.608	**65.355**	**80.093**^**1**^
Reflected Prosocial Positivity	82.624	94.087	**81.862**	**94.962**^**1**^
Direct Social Positivity	123.825	136.925	**120.556**	**132.020**
Reflected Social Positivity	134.699	144.525	**130.151**	**141.614**
Self-esteem	**233.183**	**244.646**	235.016	248.117
Self-concept clarity	203.638	216.738	**199.292**	**214.030**

*Note.* Preferred final models are depicted in bold. *AIC* Aikaike information criterion, *BIC* Bayesian information criterion. ^1^In this case AIC and BIC were inconsistent in their support for one model. Therefore, we used the sample-size adjusted BIC (ssaBIC) as an additional criterion to select the best fitting model

**Table 2:** Growth factor estimates of self-concept variables

	Growth Factors and Variance Components				
	Mean Int. (*SE*)	σ^2^	Mean LS (*SE*)	σ^2^	Mean QS (*SE*)
Dir Academic Positivity	2.67 (0.08)***	0.16**	0.10 (0.02)	0.01	
Refl Academic Positivity	2.75 (0.09)***	0.24***	0.29 (0.11)*	0.01	-0.10 (0.05)*
Dir Physical Positivity	2.91 (0.09)***	0.23**	0.20 (0.04)***	0.02	
Refl Physical Positivity	2.98 (0.09)***	0.25**	0.34 (0.11)**	0.03	-0.09 (0.05)
Dir Prosocial Positivity	3.22 (0.07)***	0.08**	0.19 (0.10)	0.01	-0.06 (0.04)
Refl Prosocial Positivity	3.15 (0.06)***	0.09**	0.26 (0.11)*	0.02*	-0.08 (0.05)
Dir Social Positivity	2.75 (0.09)***	0.21***	0.43 (0.12)***	0.05**	-0.10 (0.05)*
Refl Social Positivity	2.83 (0.09)***	0.24**	0.51 (0.12)***	0.02	-0.15 (0.06)**
Self-esteem	2.82 (0.14)***	0.52*	0.43 (0.08)***	0.17*	
Self-concept clarity	2.74 (0.09)***	0.06	-0.16 (0.19)	0.13**	0.22 (0.08)**

*Dir* Direct, *Refl* Reflected, *Int* intercept, *LS* Linear slope, *QS* Quadratic slope

*p < 0*.*05; **p < 0.01; ***p < 0.001

#### Mean level development of self-concept variables

We started out testing the trajectories of the positivity of the domain-specific self-evaluations from a *direct* perspective. For the academic domain and the physical domain, the linear model provided the best fit to the data. Only the physical domain showed a significant positive linear slope, indicating a linear increase in positivity for direct physical self-evaluations from T1 to T2 and T3. For the academic domain, the linear slope was not significant, indicating stable levels across the year. For the prosocial and social domain, quadratic growth models showed the best fit. However, similarly as for the academic domain, the model for prosocial self-evaluations did not show any significant slopes, indicating stable levels across the training year. The positivity of social self-evaluations revealed a linear increase that leveled off toward the end of training, as indicated by a positive linear and negative quadratic slope (Figs. 3a and 5a).

For all *reflected* self-evaluations, quadratic models showed the best fit. Significant linear slopes were revealed for the physical domain as well as for the prosocial domain (quadratic slopes were not significant). This indicates that the positivity of the reflected self-evaluations in these domains slightly increased in the first half of the training year. The positivity of reflected academic, and social self-evaluations both showed significant positive linear slopes and negative quadratic slopes indicating a linear increase that leveled off toward the end of training (Figs. 3b and 5a).

To test the hypothesis that the increase in the positivity of self-evaluations would be most prominent for the social domain, we saved the linear slope parameters of each participant for each domain from the LGMs, and computed a 4 (domain) x 2 (task)within-subjects factor Repeated Measures ANOVA on these estimated linear slopes. Results showed a significant interaction between domain and task (*F* (3, 111) = 13.74, *p* < 0.01, *η*^2^_p_ = 0.27). Per task, a main effect of domain was found (direct condition (*F* (3, 111) = 88.86, *p* < 0.01, *η*^2^_p_ = 0.71; reflected condition (*F* (3, 111) = 58.61, *p* < 0.01, *η*^2^_p_ = 0.61)). Post-hoc tests using Bonferroni correction revealed that in both tasks, the increase in positivity in the social domain (direct: *M* = 0.43; reflected: *M* = 0.51) was significantly larger compared to the increase in the academic domain (direct: *M* = 0.10, reflected: *M* = 0.29, both at *p* < 0.001), the physical domain (direct: *M* = 0.20, reflected: *M* = 0.34, both at *p* < 0.001), as well as the prosocial domain (direct: *M* = 0.19, reflected: *M* = 0.26, both at *p* < 0.001).

For self-esteem, the linear model provided the best fit to the data. The positive slope indicated that self-esteem showed a linear increase over time for the whole sample. For self-concept clarity, the quadratic model revealed a better fit to the data. Self-concept clarity showed to be relatively stable until halfway through the training (T1-T2), where after it increased, as indicated by a significant positive quadratic slope (Figs. 4 and 5b).

### Neural training results

#### Preregistered ROI analyses

To investigate how neural mechanisms underlying self-concept change after self-concept training, we started out by testing our pre-registered hypotheses focused on three *a priori* defined ROIs: the mPFC, Precuneus, and the right TPJ (see “Pre-registered Region of Interest (ROI) analyses” section for ROI definition). Means and standard deviations of activation in all ROIs can be found as supplementary material Table S.3. 

##### mPFC

**Fig. 6:**
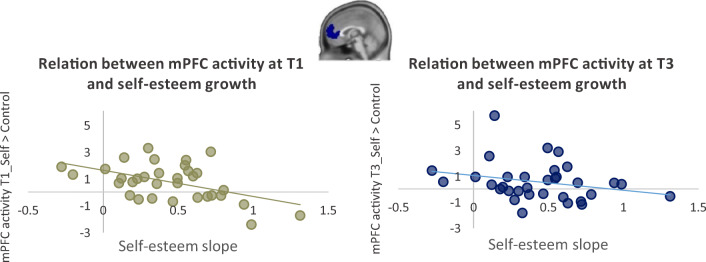
Relations between self-esteem slope and mPFC activity for the contrast self > control at each time point

First, we tested the hypothesis that increasing levels of self-esteem would be associated with larger mPFC changes, which should be evident by a time x self-esteem slope interaction. To test this effect, we performed a Repeated Measures ANOVA on the contrast self > control with Time (T1, T3) and Task (direct, reflected) as within subjects factors, the linear slope of self-esteem as covariate of interest, and age at T1 as covariate of no interest. Results showed no main effect of time (*p* = 0.400), nor an interaction between time and task (*p* = 0.237), time and self-esteem slope (*p* = 0.354), task and self-esteem slope (*p* = 0.066), or a time x task x self-esteem slope interaction (*p* = 0.973). There was however a main between-subjects effect of self-esteem slope on MPFC activity (*F* (1, 29) = 6.52 *p* = 0.016, *η*^2^_p_ = 0.18). Because the slope of self-esteem inherently contains an aspect of time (higher slopes indicate greater increases in self-esteem from T1 to T3), we checked the correlations between self-esteem slope and mPFC activity at each time point and plotted these relations for more clarity (Fig. 6). For mPFC activity at T1, the correlation with self-esteem slope was −0.49 (*p* = 0.004), indicating that participants with lower mPFC activity during self-evaluation at T1 experienced greater increases in self-esteem during their gap year. For T3, the correlation was still negative, but not significant (−0.26, *p* = 0.145). However, when comparing both correlation coefficients to each other using Fisher’s Z transformation, they did not significantly differ from each other (*z* = −1.25 *p* = 0.105). It should be noted that the correlation at T3 is difficult to interpret, whereas the correlation at T1 shows that lower mPFC activity at the first time point predicts higher self-esteem slopes.

**Fig. 7:**
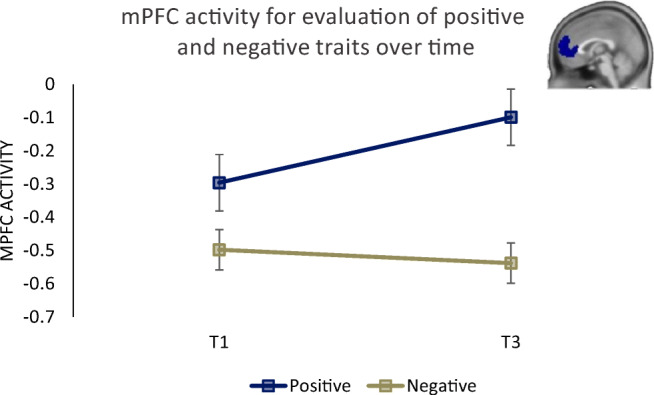
mPFC activity for evaluation of positive and negative traits over time. mPFC activity showed an increase for the evaluation of positive traits, whereas the activity for the evaluation of negative traits remained stable over time

Next, we tested time x valence effects and examined whether changes in mPFC activity varied between the evaluation of positive and negative traits. We conducted a Repeated Measures ANOVA with Time (T1, T3), Task (Direct, Reflected), and Valence (positive, negative) as within subjects factors, and age at T1 as covariate of no interest. Results showed a significant time x valence interaction (*F* (1, 30) = 7.01 *p* = 0.013, *η*^2^_p_ = 0.18). This analysis demonstrated that mPFC activity showed an increase for the evaluation of positive traits, whereas the activity for the evaluation of negative traits remained stable over time (Fig. 7). The time x task x valence interaction was not significant (*p* = 0.230), suggesting that these effects are similar for the direct and reflected task.

Lastly, we explored whether changes in mPFC activity during self-processing differed between domains with a Repeated Measures ANOVA with Time (T1, T3), Task (Direct, Reflected), and Domain (academic, physical, prosocial, social) as within subjects factors, and age at T1 as covariate of no interest. This analysis did not yield a significant time x domain interaction (*p* = 0.529), nor a time x task x domain interaction (*p* = 0.286).

##### Precuneus

Next, we tested the hypothesis that the precuneus would show increased activity over time for the evaluation of academic traits specifically. A Repeated Measures ANOVA on the contrast academic > control with Time (T1, T3) and Task (Direct, Reflected) as within subjects factors, and age at T1 as covariate of no interest did not show a significant main effect of time (*p* = 0.745), or a time x task interaction (*p* = 0.736). The addition of the slope of academic positivity (averaged across tasks) did not have a significant main effect on precuneus activity (*p* = 0.721) or an interaction with time (*p* = 0.736).

##### Right TPJ

We first examined the hypothesis that training related behavioral increases in positivity would be associated with increased right TPJ activity for direct compared to reflected self-evaluations after training. We performed a Repeated Measures ANOVA on the contrast direct > reflected (both tested vs. control condition) with Time (T1, T3) as within subjects factor, and the linear slope of a general behavioral positivity score (across domains and tasks) as covariate of interest and age at T1 as covariate of no interest. Results showed no main effect of time (*p* = 0.541), nor a main effect of the slope of overall positivity (*p* = 0.734), or an interaction between time and the slope of overall positivity (*p* = 0.725).

Next, we explored possible valence effects and tested whether training related changes in rTPJ activity varied between the evaluation of positive and negative traits. We conducted a Repeated Measures ANOVA with Time (T1, T3), Task (Direct, Reflected) and Valence (positive, negative) as within subjects factors, and age at T1 as covariate of no interest. Results showed no significant time x valence interaction (*p* = 0.818), or time x task x valence interaction (*p* = 0.346).

Finally, we explored whether training related changes in rTPJ activity during self-processing differed between domains with a Repeated Measures ANOVA with Time (T1, T3), Task (Direct, Reflected) and Domain (academic, physical, prosocial, social) as within subjects factors, and age at T1 as covariate of no interest. This analysis did not yield a significant time x domain interaction (*p* = 0.232), nor a time x task x domain interaction (*p* = 0.562).

Taken together, the results showed that change in mPFC activity was sensitive to valence, and increased more for the evaluation of positively-valenced trials than for negatively-valenced trials. Furthermore, we partly confirmed our pre-registered hypothesis that mPFC activity would be associated with self-esteem slope, but this was only confirmed for the first time point and not for change-change. The preregistered hypotheses for precuneus and rTPJ were not confirmed. Explorative whole brain analyses for the valence-based and task-based contrasts are reported in the supplement.

### Prediction results for academic and life outcomes

A final goal of this study was to examine whether individual differences in baseline and within-person changes in behavioral indices of self-concept during the gap year (T1, T2, T3) could predict outcomes related to future successful educational decision-making (T4). In line with our preregistered hypotheses, we used the LGM estimated intercepts and linear slope parameters of the variables self-esteem, self-concept clarity, academic positivity, and social positivity as predictor variables. Outcome variables were separated into two categories: one related to more *general* outcomes (satisfaction with choice, and satisfaction with life), and one related to specific *academic* outcomes (commitment to study, academic intrinsic motivation, academic and social adjustment to college, academic engagement and academic performance). Means, SDs, and correlations between the outcome variables can be found as supplementary material Table S.6. As the questions regarding academic outcomes were only asked when participants had indicated to have enrolled in higher education, the N for these variables was smaller (*N* = 22) than for the general outcome variables (*N* = 32).

In order to decrease the number of tests for the academic outcome variables, we first conducted a PCA with varimax rotation to examine whether they could also be encompassed by fewer underlying factors. Assumptions check for the PCA showed a Kaiser-Meyer-Olkin(KMO) value of 0.86, which indicated adequate sampling, and a significant Bartlett’s test of sphericity (χ^2^ = 139.035, *df* = 36, *p* < 0.001), which indicated suitability of the data for PCA. The parallel analysis indicated that two factors should be retained. The two factors together explained 75.17% of the total variance. The first factor (63.37% variance explained) contained most of the variables: the three scales related to academic engagement (vigor, dedication, absorption), the two scales related to autonomous motivation (identified regulation and intrinsic motivation), commitment to chosen study, and academic adjustment to college. The second factor consisted of social adjustment to college and academic performance. The specific factor loadings can be found in Table 3. We labeled the first factor “drive,” as it contains variables related to intrinsic motivation, engagement, and commitment to a chosen study. The factor scores were saved for each participant, so they could be used for regression analyses in SPSS. The second factor (social adjustment to college & academic performance) was more difficult to label; therefore, we chose to name this factor “Factor 2.”

**Table 3:** Factor loadings for the PCA on academic outcome variables

	Factor 1	Factor 2
Study dedication (UBES)	0.905	
Study absorption (UBES)	0.859	
Study vigor (UBES)	0.858	
Study commitment (U-MICS)	0.825	
Intrinsic motivation (SRQ)	0.743	
Academic adjustment (SACQ)	0.665	
Identified regulation (SRQ)	0.559	
Social adjustment (SACQ)		0.945
Academic performance		0.793

*Note:* Only factor loadings > 0.50 are printed in this table

Next, we performed a series of regression analyses in SPSS to test our preregistered hypotheses regarding predictions of outcome measures related to successful educational decision-making.

#### Self-esteem

Self-esteem intercept and slope did not significantly predict any academic outcomes (factor “drive,” *p* = 0.062; factor 2 (social adjustment & academic performance, *p* = 0.758)), or the general life outcomes: satisfaction of choice (*p* = 0.880) or life satisfaction (*p* = 0.657).

#### Self-concept clarity

Results revealed a significant model for Factor 2 (social adjustment and academic performance (*F* (2, 21) = 6.09, *p* < 0.001). The linear slope of self-concept clarity was positive and significant (: *β*_*slope*_ = 0.52, *p* < 0.001) indicating that individuals who showed a stronger increase in self-concept clarity over time reported better social adjustment to college and better academic performance relative to individuals with a lower SCC slope. No significant prediction models were found for other academic outcomes (factor “drive,” *p* = 0.061), or the general life outcomes: satisfaction of choice (*p* = 0.833) or life satisfaction (*p* = 0.381).

#### Social positivity

As our preregistered hypothesis regarding the social domain solely focused on predicting social adjustment to college and life satisfaction, we used social adjustment as a separate outcome variable for this analysis. Individual differences in the intercepts and slopes of the positivity of direct as well as reflected social self-evaluations were found to predict social adjustment to college (Direct: *F* (2, 21) = 4.28, *p* = 0.029; : *β*_*intercept*_ = 0.54, *p* = 0.033; *β*_*slope*_ = 0.66, *p* = 0.011; Reflected: *F* (2, 21) = 7.41, *p* = 0.004; : *β*_*intercept*_ = 0.67, *p* = 0.003; : *β*_*slope*_ = 0.64, *p* = 0.004). As both intercepts and slopes are positive, this indicates that individuals who started out with more positive social self-evaluations as well as show a stronger increase in the positivity of these self-evaluations report better social adjustment to college. No effects were found for life satisfaction (Direct: *p* = 0.156; Reflected *p* = 0.223).

#### Academic positivity

Following our preregistered hypotheses regarding the influence of individual differences in starting points and trajectories of positivity in the academic domain, we only focused on academic motivation, academic adjustment, and academic performance as separate outcome variables. Intercept and slopes of the positivity of direct or reflected academic self-evaluations did not predict academic motivation (Direct: *p* = 0.240; Reflected *p* = 0.294) or academic adjustment (Direct: *p* = 0.377; Reflected *p* = 0.307). For academic performance, the ANOVA model of both direct and reflected academic positivity was significant (Direct: *F* (2, 22) = 4.89, *p* = 0.019; Reflected *F* (2, 22) = 6.37, *p* = 0.007). However, for both models, the individual coefficients of both intercept and slope were not significant.

## Discussion

This study tested the effects of a naturalistic self-concept training within a gap year context on behavioral and neural correlates of self-evaluations, as well as the long-term effects for educational decision-making. The study resulted in four main findings. First, the 1-year training period was associated with increases in self-esteem, self-concept clarity, and positivity of domain-specific self-evaluations. Changes were largest for social self-evaluations, consistent with the notion that the social self is an important aspect of our identity (Pfeifer & Berkman, 2018). Second, participants with lower medial PFC activity before training showed larger self-esteem increases over the year. Third, brain activity in medial PFC, an important region for self-evaluation(Denny et al., 2012), increased more for the evaluation of positive self-traits than for negative self-traits. Finally, individual differences in changes in self-concept clarity and social self-evaluations, but not self-esteem, positively predicted outcomes related to future-oriented educational choices. The discussion is organized along the lines of these four main findings.

### Behavioral correlates of a naturalistic gap year program

An emerging problem in our society is that it is challenging for young people to find an educational program which matches their self-views, which as a result can lead to high levels of drop-out and an increasing number of gap years (Dutch Ministry of Education, 2018a, 2018b). The group of adolescents who experience these difficulties is large and heterogeneous, and some of them may actively seek out help. In this study, our goal was to examine whether adolescents who decide to take part in a naturalistic self-concept training program would show positive changes in domain-specific self-evaluations, self-esteem, and self-concept clarity. These hypotheses were based on a recent study in which we showed that self-esteem and self-concept clarity were significantly lower in adolescents who experienced difficulties with educational decision-making compared with peers who already successfully transitioned into higher education (van der Aar et al., 2019a). As predicted, participants who took part in this program showed increased levels of self-esteem, self-concept clarity, and positivity of self-evaluations across all domains (academic, (pro)social, and physical appearance) after the program. Notably, changes were most significant for social self-evaluations, suggesting that the difficulties within this group may be broader than academic decision-making and may reflect a general difficulty with fitting in (Di Fabio et al., 2013).

More specific analyses were conducted to examine time-related transitions by including an additional half-way time point. These analyses revealed that especially for *reflected*self-concept (“peers think about me that I am…”), changes occurred mostly in the first period of the program. In this period, the focus of the program was on “me” and “others,” possibly indicating that these modules have a larger impact on self-evaluation from perspectives of others. Also, the start of the training within a group setting with same-aged peers could have had a direct positive effect on these reflected self-evaluations(Forsyth, 2015). Interestingly, self-concept clarity showed a change only in the second half of the program, which had a stronger focus on “travel” and “world,” suggesting that self-concept clarity increases more in interaction with new outside perspectives. Another reason for this relatively late increase in self-concept clarity could be that it takes more time for self-reflection and reconsideration to develop an increasingly clear and coherent self-concept. These patterns were different from changes in self-esteem, which as expected increased gradually over the course of the program.

To date, research on the effects of taking a gap year between high school and higher education have shown mixed results. For example, some studies have found positive effects with regard to personal development, such that gap-year takers felt more confident, mature, and independent after their gap year (King, 2011). Beneficial effects also have been found for academic outcomes, such as increased academic motivation for gap-year takers (Martin, 2010). On the contrary, others have found negative outcomes for attainment (e.g., gap-year students were less likely to start a major or more likely to drop out of a university degree) or showed no significant benefits in relation to goal engagement and self-confidence(Parker et al., 2015). The gap years examined in these studies often were unstructured (consisting of traveling or working), and it is unclear what the mechanisms were that ultimately would help adolescents with their decision-making process afterwards.

The current study evaluated changes in relation to a relatively structured gap year program in which participants followed specific modules targeted at improving self-esteem and self-concept clarity. These first results seem promising regarding the malleability of self-concept during late adolescence. Moreover, they complement existing intervention programs, which are mostly based on elementary to high schools, by its focus on the transitional phase of late adolescence/emerging adulthood and specifically to the context of future educational decision-making. However, it is important to note that the naturalistic design of the Gap Year program was inherently not (quasi) experimental, and we were not able to include a suitable control group, because waiting list participants often seek out alternatives in the intended gap year and were not available for two fMRI sessions. Therefore, the findings should be interpreted with caution. For example, we cannot conclude whether those who decide to engage in a gap-year program are altogether similar in several characteristics, including self-concept (malleability), compared with those who more informally take a gap year or drop out of college without returning to a structured program. Therefore, these findings should be strengthened in a future design that includes a control group of adolescents who take a gap year that does not take place within a structured program. Importantly, a recent study of research institute Noorda and Co (2019) examining a different cohort of the Gap Year program (2018-2019) with the same sample size and accompanied by a control group found similar beneficial effects for adolescents’ self-concept development and future orientation that were significantly larger for Gap Year participants. Additionally, tentative comparisons with existing longitudinal studies showed that changes in self-esteem and self-concept clarity were larger compared with what is usually reported in this age range. For example, in a 4-wave longitudinal study, von Soest et al. (2016) reported an average increase (slope) in global self-esteem of 0.13 between the ages 13–31 years compared with a linear increase of 0.43 in this sample. Similarly, self-concept clarity showed only minor increases in the period of late adolescence/early adulthood (slope of 0.03) according to a 6-wave longitudinal study of Crocetti et al. (2016), whereas our gap year participants showed an average increase of 0.22. However, future replications among different samples and across different age periods is warranted.

### Neural correlates of a naturalistic gap year program

Several studies have demonstrated that a network of brain regions that is often implicated in social brain processing, including the medial PFC, PCC/precuneus, and TPJ also are involved in self-evaluations(Pfeifer & Berkman, 2018). Specifically, the medial PFC often is implicated as an important region for mentalizing about self and others (Denny et al., 2012). This study tested the changes in neural activity in these regions during the direct and reflected evaluations of self-traits that could be positive or negative and that could be targeted to academic, (pro)social, and physical appearance domains. Results from the first time point were previously published (van der Aar et al., 2019a). These findings showed that mPFC activity was correlated with self-esteem, such that individuals with higher self-esteem showed more activity in medial PFC during self-evaluation. The current study demonstrated that mPFC activity at the first time point predicted self-esteem*change*. That is, individuals who were already high in medial PFC activity during self-evaluation showed no large change in self-esteem, whereas participants who were low in medial PFC activity showed the largest increase in self-esteem. These findings extend our previous suggestion that self-esteem is an important prerequisite for self-evaluations and associated mPFC activity. It should be noted that we could not confirm the hypothesis of an mPFC change and self-esteem change correlation. Longitudinal, experimental studies, including larger samples, may unravel the time-related relations between mPFC activity during self-evaluations and self-esteem.

Previous findings also showed that mPFC is more strongly recruited for positive self-evaluations, possibly because these often are interpreted as more applicable to self (Cosme et al., 2019; D’Argembeau, 2013; Moran et al., 2006). Consistent with this finding, we demonstrated that mPFC activity was higher for the evaluation of positive than negative self-traits(see also Van der Cruijsen et al., 2018) but also that activity increased more for positive than negative self-traits after the training compared to before the training. These findings fit well with the general increase in behavioral positivity ratings, possibly reflecting that positive traits were considered more applicable to self (D’Argembeau, 2013). However, similar to the behavioral results, these neural findings related to the mPFC should be validated in future studies, including a control group.

In addition to the valence effects, we also preregistered the predictions that PCC/precuneus activity associated with academic self traits would be correlated with increased positivity of academic self-evaluations(based on van der Aar et al., 2019b) and that right TPJ activity associated with reflected self-evaluations would be correlated with increased general positivity of self-traits(based on Van der Cruijsen et al., 2019). Both of these predictions were not confirmed. One possible explanation is that the variance for the behavioral positivity score of self-evaluations was relatively low to reveal correlations with neural activity. Adolescents showed significant individual differences in developmental trajectories of self-esteem and self-concept clarity, whereas these individual differences were less pronounced for domain-specific self-evaluations. Thus, possibly the lack of individual differences together with the relatively small sample size did not allow us to detect the predicted brain-behavior correlations for self-evaluations in these regions. Alternatively, self-related activity in precuneus and right TPJ may be less changeable within the age-group of this study (late adolescents/young adults), or changes in mPFC activity are more detectable. However, these interpretations are speculative and future studies including larger samples should formally test these hypotheses.

### Predicting educational decision-making

An important question in training research concerns whether changes related to training are predictive of future real-life outcomes. The educational outcomes in this study were separated in general positive outcomes (satisfaction with choice, and life satisfaction), and outcomes specifically related to an academic context (factors “drive” and “social adjustment/academic performance”). These findings showed separable effects for self-esteem and self-concept clarity. That is, even though self-esteem increased gradually during the program, it did not significantly predict any of the outcome measures. In contrast, larger increases in self-concept clarity positively predicted social adjustment to college and academic performance. Interestingly, self-concept clarity increased relatively late in the program. Possibly, self-concept clarity change needs a longer investment but also shows larger long-term effects. An opportunity for future research is to further examine the direct or indirect role of self-concept clarity in the prediction of positive educational outcomes in higher education. For example, increases in self-concept clarity could lead to a better suited choice of education accompanied by meeting other students with similar interests, leading to better social adjustment. This social adjustment could subsequently improve academic performance through increased collaboration and help from others.

Consistent with our hypotheses, a second predictor for social adjustment to college was the intercept and change in the positivity of social self-evaluations. It is interesting to note that the predictions mostly concerned social adjustment to college and not academic outcomes related to commitment, motivation, or adjustment (“drive”), suggesting that different factors might affect drive. Future studies could test, for example, whether “academic drive” is more strongly predicted by processes, such as (growth)mind-sets rather than self-concept(Burnette et al., 2013). Overall, the results emphasize that an important focus of the gap year training is related to “social factors,” such as improving social skills, working together, giving and receiving feedback, and training within a group setting. This is consistent with the finding that the strongest increases were found for positivity scores in the social domain and that predictions were mostly related to the social aspects of adjusting to the chosen college. Making successful future-oriented educational choices can be considered a process that should not only have a focus on academic skills but includes an important role for social development as well.

### Limitations and future directions 

This study has several strengths, including the use of a 4-wave longitudinal design with both behavioral and fMRI assessments, which allowed us to investigate the effects of self-concept training on multiple levels as well as predict individual differences in future educational decision-making in this unique group of adolescents. Although this longitudinal design increases power, there are still some limitations regarding the sample size. For example, although our sample size is considered sufficient with respect to fMRI research (Geuter et al., 2018), it was too small to test for additional important differences in self-concept development between categorical variables, such as gender. Additionally, given that the experience of starting and subsequently dropping out of a study program may significantly impact the development of self-concept, and academic and social outcomes, it also would be an important future direction to further examine possible differences between these subgroups, such as whether or not adolescents started their gap year directly after high school. Future studies would benefit from including these additional variables and testing relations with educational decision-making in larger samples, using longitudinal designs.

A second strength of this study was the use of the naturalistic Gap Year program, which involved individuals who were intrinsically motivated to participate in the training. At the same time, this naturalistic environment limited us in finding a suitable control group. Future studies should include a control group of adolescents who take a gap year that does not take place within a structured program, to validate the current findings and optimize generalizability.

Taken together, this study using a naturalistic program within a gap year context showed that training focused on the self enhanced multiple aspects of self-concept (self-esteem, self-concept clarity, and positivity of domain-specific self-evaluations) and associated activity in mPFC related to positive self-evaluations. This study aimed to speak to an emerging problem with an increasingly higher number of adolescents taking gap years before starting higher education. According to Researchned (2018), this increase could be a result of the introduction of the Dutch student loan system, which increased the pressure for adolescents to make the “right” decision for their future straight away, because dropping out or switching between programs could lead to significantly high costs. As a consequence, many adolescents point out they suffer from choice overload and are afraid to make a wrong decision. However, this problem is not limited to the Netherlands and reflects a broader tendency of students experiencing burnout due to various societal pressures (Lin & Huang, 2014). This study was conducted before the COVID-19 crisis, but this may be a new societal challenge that could affect adolescents’ process of choosing a well-suited future educational career path, although the long-term effects remain to be investigated.

## Conclusions

This study showed that for late adolescents self-concept training can lead to increased positive domain-specific self-evaluations, self-esteem, and self-concept clarity, and eventually better social adjustment to college and academic performance. Although these results were obtained within a gap-year context, the positive outcomes point toward the implication of increasing the focus on self-concept development already early in high school to help adolescents better understand their traits, interests, abilities, and goals, thereby increasing their chances of finding a suitable major.

## Supplementary Information

ESM 1 (DOCX 3990 kb)
