# Supplementary material for: Better self-concept, better future choices? Behavioral and neural changes after a naturalistic self-concept training program for adolescents
Source: Cogn Affect Behav Neurosci. 2021 Sep 27;22(2):341–61. doi: 10.3758/s13415-021-00946-1 (PMC8475836; doi:10.3758/s13415-021-00946-1)
Supplement: Supplementary file 1 — (DOCX 3990 kb) [file 13415_2021_946_MOESM1_ESM.docx]

**Supplementary material**

**Table S.1**

|  | Total Sample  (*N* = 38) | Dropped out of Gap Year program  (*N* = 2) | | Started directly after high school  (*N* = 23) | Started directly after high school  (*N* = 15) |
| --- | --- | --- | --- | --- | --- |
|  | *M (SD)* | | | | |
| Age (years) | 18.73 (1.47) | 17.81 (0.57) | | 18.12 (0.76) | 19.67 (1.79) |
| IQ | 104.47 (9.5) | 103.75 (1.77) | | 104.57 (9.90) | 104.33 (9.18) |
| RCADS total | 38.32 (21.28) | 47.50 (36.06) | | 35.60 (21.78) | 42.47 (20.51) |
| Social Phobia  Panic Disorder  MDD  SAD  GAD  OCD | 12.66 (7.18) | 15.00 (15.56) | | 12.48 (8.02) | 12.93 (5.91) |
|  | 4.95 (3.96) | 3.50 (0.70) | | 3.61 (2.89) | 7.00 (4.57) |
|  | 8.32 (5.34) | 11.00 (7.07) | | 7.39 (4.81) | 9.73 (5.95) |
|  | 3.18 (2.99) | | 7.00 (5.66) | 3.35 (3.24) | 2.93 (2.66) |
|  | 5.34 (3.58) | | 6.50 (6.36) | 5.04 (3.82) | 5.80 (3.26) |
|  | 3.87 (3.12) | | 4.50 (2.12) | 3.74 (3.40) | 4.01 (2.74) |
| SDQ total difficulties | 11.42 (4.46) | | 9.00 (1.41) | 12.13 (4.46) | 10.33 (4.40) |
| Emotional Problems  Conduct Problems  Hyperactivity score  Peer Problems | 3.89 (2.69) | | 2.00 (.00) | 4.52 (2.81) | 2.93 (2.25) |
|  | 1.16 (1.20) | | 1.00 (.00) | 1.17 (1.23) | 1.13 (1.19) |
|  | 3.92 (2.05) | | 4.00 (.00) | 3.96 (1.99) | 3.87 (2.20) |
|  | 2.45 (1.41) | | 2.00 (1.41) | 2.48 (1.41) | 2.40 (1.45) |
| Clinical diagnoses |  | |  |  |  |
| ADHD  ADD  ASS  Depression | 2  3  1  1 | | 0  0  0  0 | 0  0  1  1 | 2  3  0  0 |

Demographics and report of clinical symptoms and diagnoses of the whole sample and sub-groups

*Note*: RCADS = Revised Children’s Anxiety and Depression Scale (range total score 0 - 141). MDD = Major Depressive Disorder; SAD = Separation Anxiety Disorder; GAD = Generalized Anxiety Disorder; OCD = Obsessive Compulsive Disorder. SDQ = Strengths and Difficulties Questionnaire (range total score 0 – 40, scale score 1 – 10).

**Table S.2**

Means and Standard Deviations of all behavioral self-concept variables

| *Variable* | *M* | *SD* |
| --- | --- | --- |
| 1.Dir Academic Positivity T1 | 2.69 | .56 |
| 2.Dir Academic Positivity T2 | 2.80 | .46 |
| 3.Dir Academic Positivity T3 | 2.89 | .46 |
| 4.Refl Academic Positivity T1 | 2.75 | .55 |
| 5.Refl Academic Positivity T2 | 3.36 | .44 |
| 6.Refl Academic Positivity T3 | 2.95 | .59 |
| 7.Dir Physical Positivity T1 | 2.93 | .56 |
| 8.Dir Physical Positivity T2 | 3.13 | .41 |
| 9.Dir Physical Positivity T3 | 3.32 | .44 |
| 10.Refl Physical Positivity T1 | 2.98 | .54 |
| 11.Refl Physical Positivity T2 | 3.26 | .48 |
| 12.Refl Physical PositivityT3 | 3.33 | .39 |
| 13.Dir Prosocial Positivity T1 | 3.24 | .43 |
| 14.Dir Prosocial Positivity T2 | 3.34 | .33 |
| 15.Dir Prosocial Positivity T3 | 3.35 | .34 |
| 16.Refl Prosocial Positivity T1 | 3.15 | .38 |
| 17.Refl Prosocial Positivity T2 | 3.36 | .44 |
| 18.Refl Prosocial Positivity T3 | 3.37 | .40 |
| 19.Dir Social Positivity T1 | 2.69 | .54 |
| 20.Dir Social Positivity T2 | 3.07 | .40 |
| 21.Dir Social Positivity T3 | 3.22 | .44 |
| 22.Refl Social Positivity T1 | 2.83 | .57 |
| 23.Refl Social Positivity T2 | 3.19 | .49 |
| 24.Refl Social Positivity T3 | 3.23 | .48 |
| 25.Dir Overall Positivity T1 | 2.88 | .35 |
| 26.Dir Overall Positivity T2 | 3.08 | .26 |
| 27.Dir Overall Positivity T3 | 3.19 | .31 |
| 28.Refl Overall Positivity T1 | 2.93 | .39 |
| 29.Refl Overall Positivity T2 | 3.20 | .39 |
| 30.Refl Overall Positivity T3 | 3.22 | .37 |
| 31.Self-esteem T1 | 2.83 | .88 |
| 32.Self-esteem T2 | 3.22 | .76 |
| 33.Self-esteem T3 | 3.69 | .63 |
| 34.Self-concept clarity T1 | 2.74 | .55 |
| 35.Self-concept clarity T2 | 2.82 | .60 |
| 36.Self-concept clarity T3 | 3.31 | .75 |

*Note*. Dir=Direct; Refl= Reflected.

Range of scores self-concept domains 1-4; self-esteem and self-concept clarity 1-5

**Table S.3**

Means and Standard Deviations of ROI activation

| *ROI* | *M* | *SD* |
| --- | --- | --- |
| ***mPFC (*** *x = 6, y = 59, z = 13)* |  |  |
| T1_Self Direct | -0.26 | 1.17 |
| T1_Self Direct Positive | -0.11 | 1.25 |
| T1_Self Direct Negative | -0.40 | 1.14 |
| T1_Self Direct Academic | -0.31 | 1.40 |
| T1_Self Direct Physical | -0.20 | 1.28 |
| T1_Self Direct Prosocial | -0.43 | 1.31 |
| T1_Self Direct Social | -0.34 | 1.22 |
| T1_Self Reflected | -0.42 | 0.74 |
| T1_Self Reflected Positive | -0.36 | 0.84 |
| T1_Self Reflected Negative | -0.48 | 0.70 |
| T1_Self Reflected Academic | -0.59 | 1.13 |
| T1_Self Reflected Physical | -0.19 | 1.14 |
| T1_Self Reflected Prosocial | -0.68 | 0.91 |
| T1_Self Reflected Social | -0.45 | 1.04 |
| T1_Control | -1.11 | 1.04 |
| T3_Self Direct | -0.26 | 0.73 |
| T3_Self Direct Positive | -0.06 | 0.90 |
| T3_Self Direct Negative | -0.46 | 0.72 |
| T3_Self Direct Academic | -0.36 | 0.99 |
| T3_Self Direct Physical | -0.13 | 0.72 |
| T3_Self Direct Prosocial | -0.70 | 0.79 |
| T3_Self Direct Social | -0.05 | 0.83 |
| T3_Self Reflected | -0.28 | 0.89 |
| T3_Self Reflected Positive | -0.04 | 0.82 |
| T3_Self Reflected Negative | -0.51 | 1.04 |
| T3_Self Reflected Academic | -0.59 | 0.92 |
| T3_Self Reflected Physical | -0.12 | 0.84 |
| T3_Self Reflected Prosocial | -0.47 | 1.01 |
| T3_Self Reflected Social | -0.18 | 1.14 |
| T3_Control | -0.98 | 1.53 |
| ***Precuneus***  *(x = -9, y = -52, z = 28)* |  |  |
| T1_Self Direct Academic | 1.13 | 1.97 |
| T1_Self Reflected Academic | 1.45 | 1.67 |
| T1_Control | 0.05 | 2.07 |
| T3_Self Direct Academic | 1.41 | 1.62 |
| T3_Self Reflected Academic | 1.17 | 1.76 |
| T3_Control | 0.40 | 2.21 |
| ***Right TPJ (*** *x = 60, y = -28, z = 46)* |  |  |
| T1_Self Direct | 0.55 | 1.12 |
| T1_Self Direct Positive | 0.58 | 1.18 |
| T1_Self Direct Negative | 0.52 | 1.11 |
| T1_Self Direct Academic | 0.55 | 1.32 |
| T1_Self Direct Physical | 0.63 | 1.08 |
| T1_Self Direct Prosocial | 0.53 | 1.28 |
| T1_Self Direct Social | 0.50 | 1.24 |
| T1_Self Reflected | 0.27 | 1.10 |
| T1_Self Reflected Positive | 0.31 | 1.14 |
| T1_Self Reflected Negative | 0.24 | 1.15 |
| T1_Self Direct Academic | 0.10 | 1.32 |
| T1_Self Direct Physical | 0.47 | 1.00 |
| T1_Self Direct Prosocial | 0.37 | 1.32 |
| T1_Self Direct Social | 0.15 | 1.27 |
| T1_Control | -0.36 | 1.59 |
| T3_Self Direct | 0.52 | 1.12 |
| T3_Self Direct Positive | 0.44 | 1.27 |
| T3_Self Direct Negative | 0.59 | 1.11 |
| T3_Self Direct Academic | 0.43 | 1.26 |
| T3_Self Direct Physical | 0.59 | 1.15 |
| T3_Self Direct Prosocial | 0.55 | 1.16 |
| T3_Self Direct Social | 0.50 | 1.29 |
| T3_Self Reflected | 0.33 | 1.13 |
| T3_Self Reflected Positive | 0.43 | 1.16 |
| T3_Self Reflected Negative | 0.24 | 1.22 |
| T3_Self Reflected Academic | 0.16 | 1.15 |
| T3_Self Reflected Physical | 0.49 | 1.36 |
| T3_Self Reflected Prosocial | 0.29 | 1.21 |
| T3_Self Reflected Social | 0.39 | 1.28 |
| T3_Control | -0.17 | 1.60 |

*Note:* Task and valence conditions are averaged across domains. The domain conditions are averaged across valences.

Whole-brain analyses

In addition to our pre-registered ROI analyses, we also examined the valence contrast positive > negative on a whole brain level. One sample t-tests showed that at each time point and across tasks, evaluating positive versus negative traits resulted in activity in the mPFC, ACC, PCC/Precuneus, Hippocampus, and Angular Gyrus (**Figure S.1A, S.1B, Table S.4**). We conducted a Flexible Factorial ANOVA with time (T1, T3) and valence (positive, negative) to investigate possible increases in activation over time. The main effect of time (T3 > T1) revealed activation in the right putamen, right insula, and left superior temporal gyrus (**Figure S.1C, Table S.4**).

Finally, on a whole-brain level, we explored changes in task-based effects for both the direct and reflected task, tested versus the control condition. For the contrast Direct > Control on T1, activity was shown in the mPFC, ACC, and TPJ. On T3, activity was only observed in the SMA (see **Figure S.2A, S.2B and Table S.5**). No differences were found between time points. For the contrast Reflected > Control, activity was observed in the lingual gyrus on both time points. On T3, activity was also shown in the SMA (see Figure **S.3A, S.3B and Table S.5**). A Flexible Factorial ANOVA with time (T1, T3) and condition (reflected, control) revealed additional increases in the left middle- and posterior cingulate cortex (MCC and PCC). However, the extracted ROI of this cluster indicated that this effect was mostly driven by increases in activation for the control condition (Figure **S.3C. and Table S.5**).


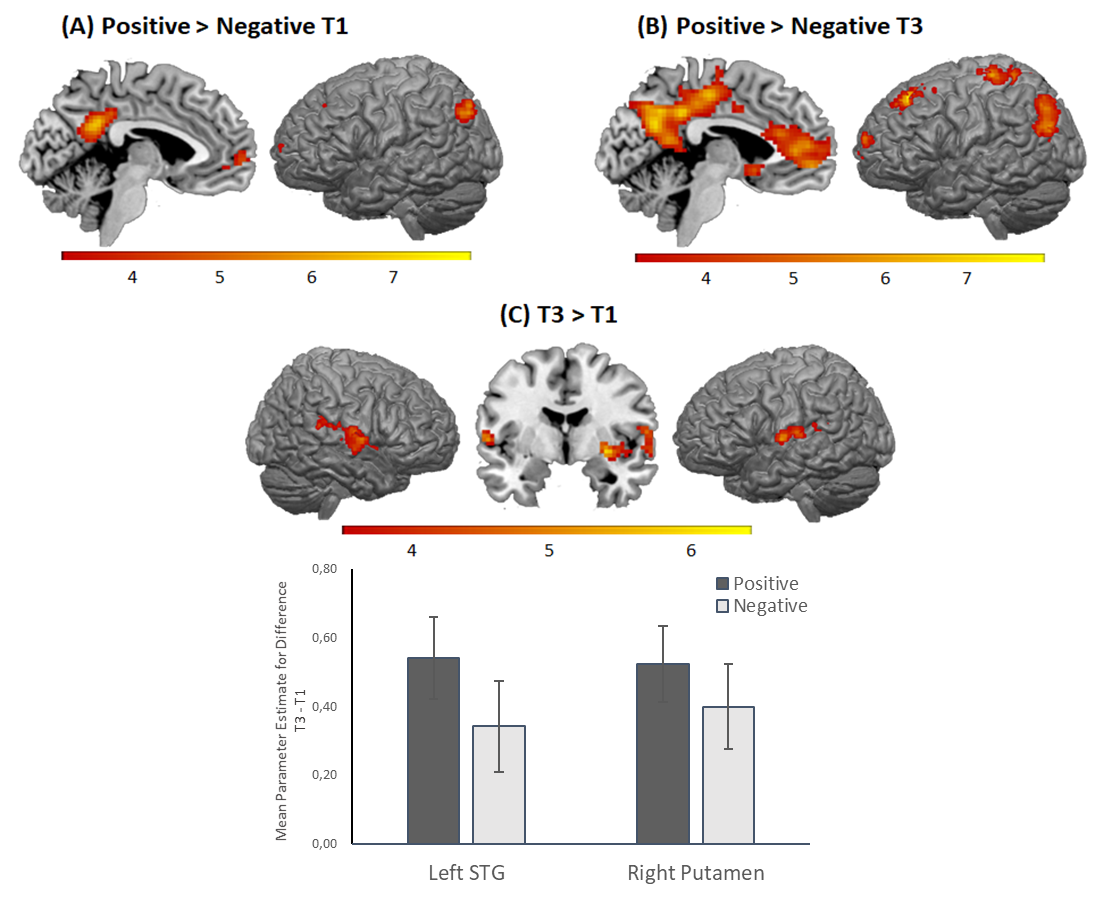


**Figure S.1:** The contrast positive > negative (both tasks combined) resulted on both time points in activity in the mPFC, ACC, PCC/Precuneus, Hippocampus, and Angular Gyrus. A significant increase in activation for T3 > T1 was observed in right putamen and left superior temporal gyrus (STG). All regions survived FDR-cluster correction (*p* < .05) at an initial uncorrected threshold of *p* < 0.001.

**Table S.4**

Regions activated during the valence contrast positive > negative at each time point separately (T1, T3) and for T3 > T1

| *Region* | | *BA* | *Coordinates* | | | *Cluster Size* | *T* |
| --- | --- | --- | --- | --- | --- | --- | --- |
| *Pos > Neg T1* |  |  |  |  |  |  |  |
|  | L Posterior Cingulate Cortex | 23 | -6 | -49 | 25 | 333 | 6.90 |
|  | R Precuneus | 23 | 9 | -52 | 28 |  | 5.43 |
|  | L Middle Cingulate Cortex | 23 | 0 | -37 | 37 |  | 4.96 |
|  | L Superior Medial Gyrus | 10 | -3 | 62 | 1 | 120 | 4.32 |
|  | R Anterior Cingulate Cortex | 10 | 6 | 47 | 7 |  | 4.18 |
|  | L Mid Orbital Gyrus | 32 | 0 | 50 | -5 |  | 3.74 |
|  | Hippocampus | 36 | -24 | -22 | -17 | 74 | 4.63 |
|  | L Hippocampus | 54 | -36 | -28 | -14 |  | 4.33 |
|  | L Parahippocampal Gyrus | 36 | -18 | -34 | -11 |  | 4.17 |
|  | L Angular Gyrus | 39 | -39 | -76 | 43 | 67 | 5.33 |
|  | L Middle Occipital Gyrus | 39 | -45 | -73 | 37 |  | 5.31 |
|  | L Superior Frontal Gyrus | 8 | -18 | 26 | 46 | 38 | 4.73 |
|  | L Middle Frontal Gyrus |  | -21 | 20 | 52 |  | 4.53 |
|  |  |  |  |  |  |  |  |
| *Pos > Neg T3* |  |  |  |  |  |  |  |
|  | L Superior Frontal Gyrus | 8 | -18 | 35 | 49 | 218 | 7.38 |
|  | L Middle Frontal Gyrus | 8 | -30 | 20 | 49 |  | 3.88 |
|  | L Precuneus | 31 | -15 | -61 | 31 | 1678 | 7.07 |
|  | L Middle Cingulate Cortex | 23 | 0 | -43 | 34 |  | 6.96 |
|  | L Precuneus | 31 | 0 | -61 | 28 |  | 6.91 |
|  | R Middle Orbital Gyrus | 10 | 6 | 53 | -2 | 813 | 6.45 |
|  | R Anterior Cingulate Cortex | 32 | 6 | 29 | 19 |  | 5.77 |
|  | L Anterior Cingulate Cortex | 32 | -3 | 41 | 7 |  | 5.73 |
|  | L Postcentral Gyrus | 4 | -36 | -31 | 61 | 308 | 6.15 |
|  | L Superior Parietal Lobe | 7 | -27 | -46 | 67 |  | 5.02 |
|  | L Postcentral Gyrus |  | -21 | -28 | 64 |  | 4.62 |
|  | L Angular Gyrus | 39 | -54 | -70 | 25 | 289 | 5.78 |
|  | L Middle Temporal Gyrus | 39 | -42 | -61 | 22 |  | 5.42 |
|  | L Angular Gyrus |  | -51 | -67 | 40 |  | 5.33 |
|  | L Parahippocampal Gyrus | 37 | -24 | -31 | -17 | 60 | 4.83 |
|  | L Fusiform Gyrus | 37 | -30 | -43 | -20 |  | 4.28 |
|  | L Cerrebellum |  | -18 | -46 | -17 |  | 3.53 |
|  | L Caudate nucleus | 48 | -6 | 11 | -8 | 65 | 4.78 |
|  |  |  |  |  |  |  |  |
| *PosNeg T3 > T1* |  |  |  |  |  |  |  |
|  | R Putamen | 49 | 33 | -13 | -8 | 410 | 6.01 |
|  | R Putamen | 49 | 30 | -4 | -5 |  | 5.50 |
|  | R Insula |  | 33 | -25 | 28 |  | 5.13 |
|  | L Rolandic Operculum | 1 | -42 | -22 | 19 | 201 | 5.17 |
|  | L Superior Temporal Gyrus | 6 | -57 | -4 | 4 |  | 4.73 |
|  | L Superior Temporal Gyrus | 22 | -45 | -40 | 16 |  | 4.55 |

*Note.* Names were based on the Automatic Anatomical Labeling (AAL) atlas.


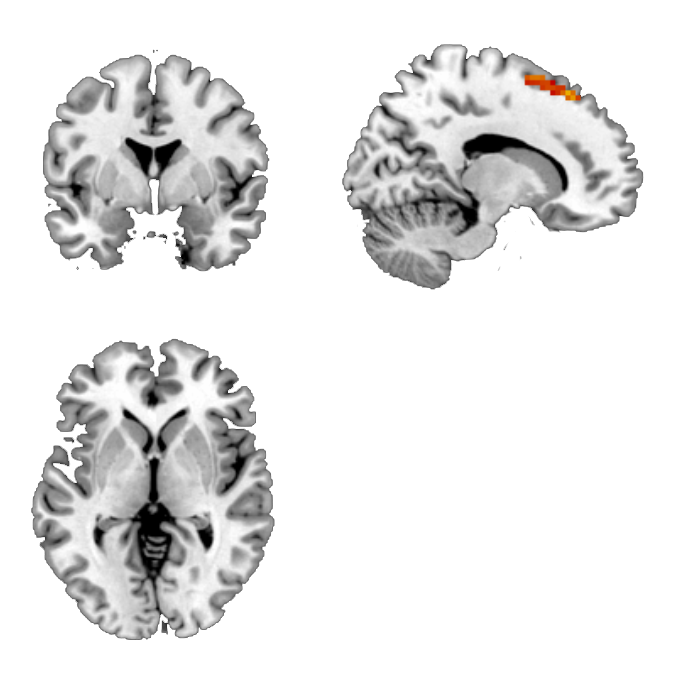


**(B) Direct > Control T3**

**(A) Direct > Control T1**


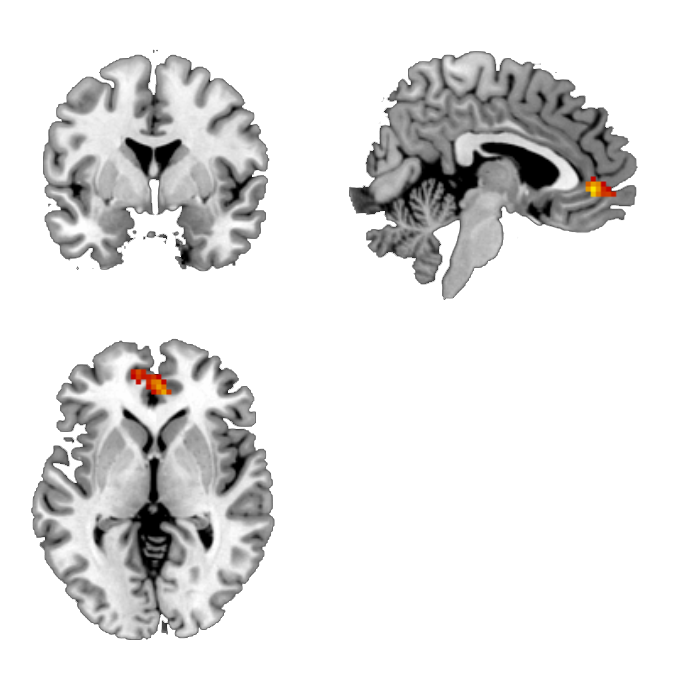

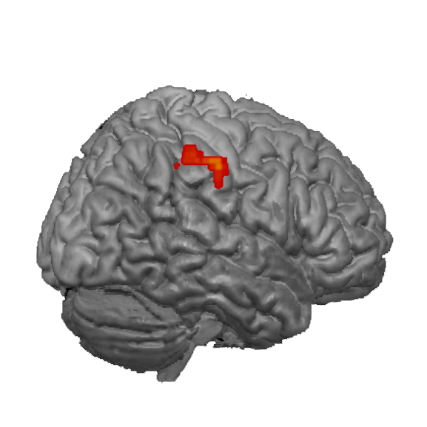

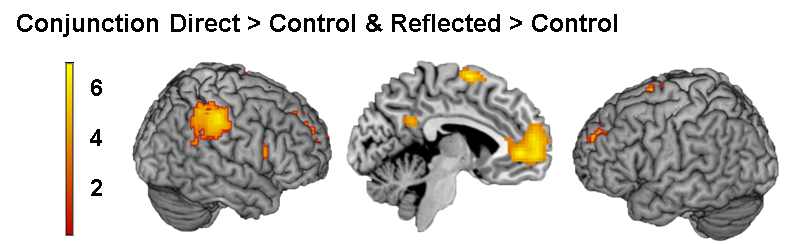


3 4 5 6


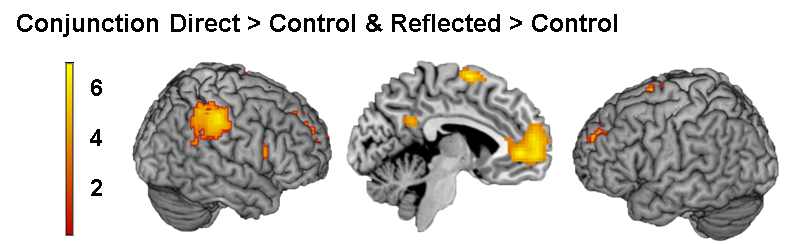


3 4 5

**Figure S.2:** The task based whole brain contrast direct > control resulted on T1 in activity in the mPFC, ACC, and TPJ. On T3 activity was only observed in the SMA. No differences between time-points were observed. All regions survived FDR-cluster correction (*p* < .05) at an initial uncorrected threshold of *p* < 0.001.


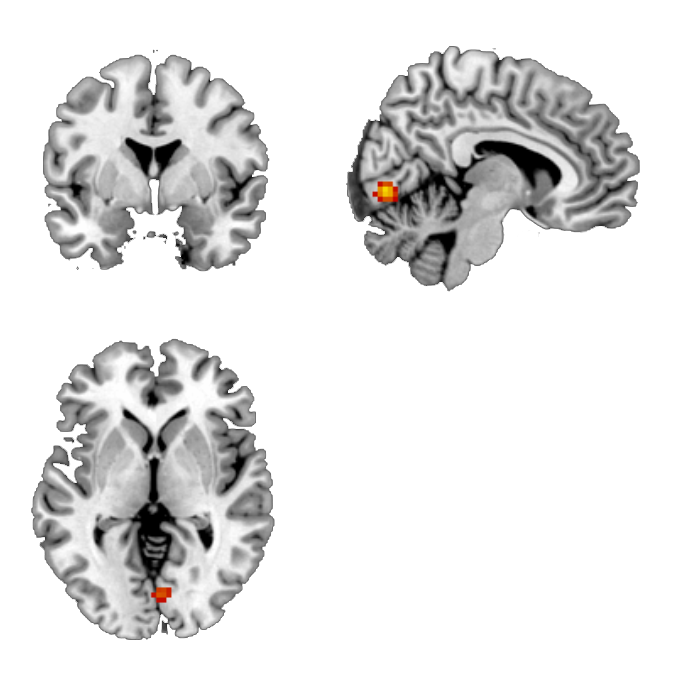

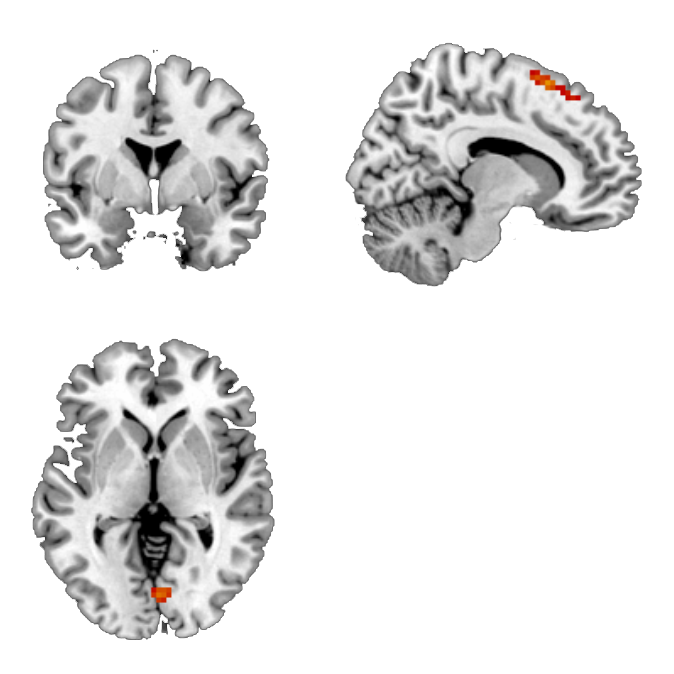

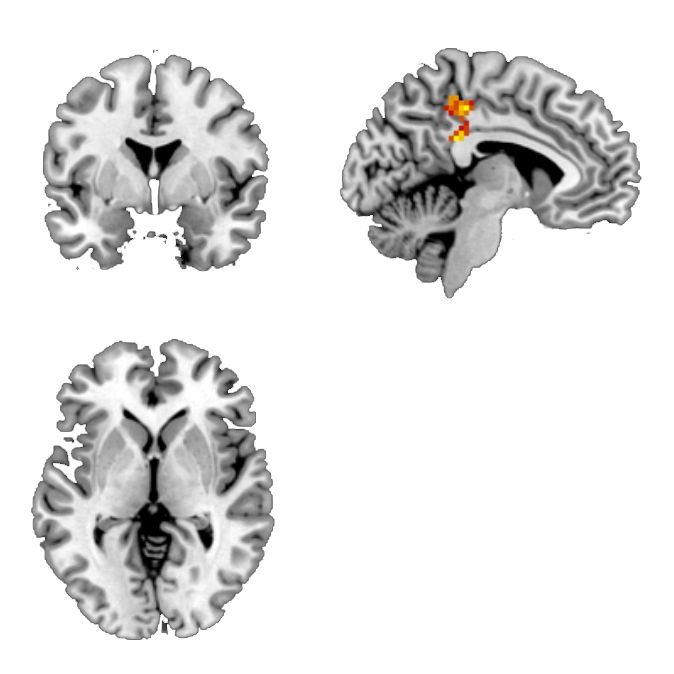


**(C) T3 > T1**

**(A) Reflected > Control T1**

**(B) Reflected > Control T3**


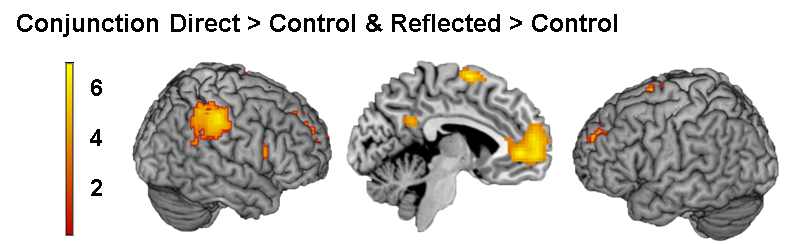


4 5 6


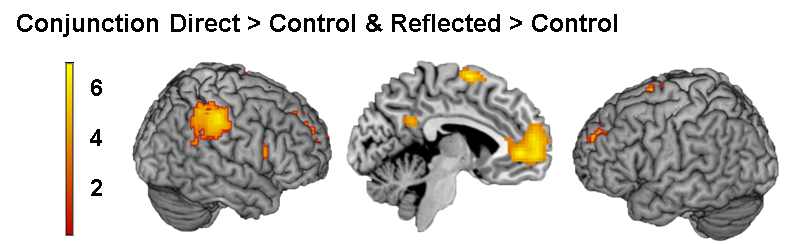


4 5 6


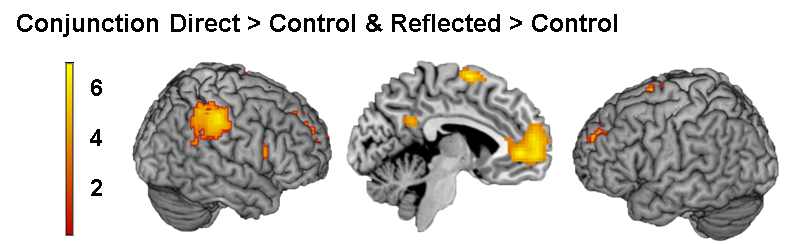


3 4 5


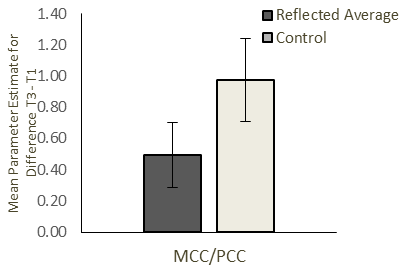


**Figure S.3**: The task based whole brain contrast reflected > control resulted on T1 in activity in the lingual gyrus. On T3 activity was additionally observed in the SMA. A significant increase in activation for T3 > T1 was shown in the left MCC and PCC. All regions survived FDR-cluster correction (*p* < .05) at an initial uncorrected threshold of *p* < 0.001

**Table S.5**

| *Region* | | *BA* | *Coordinates* | | | *Cluster Size* | *T* |
| --- | --- | --- | --- | --- | --- | --- | --- |
| *Self > Control T1* |  |  |  |  |  |  |  |
|  | L Anterior Cingulate Cortex (ACC) | 32 | 6 | 41 | -2 | 80 | 5,78 |
|  | L Mid Orbital Gyrus | 10 | -9 | 53 | -2 |  | 4,78 |
|  | L Middle Cingulate Cortex (MCC) | 32 | -9 | 44 | -2 |  | 4,31 |
|  | R Supramarginal Gyrus (TPJ) | 40 | 63 | -25 | 43 | 58 | 4,46 |
|  | R Inferior Parietal Lobe (IPL) | 40 | 45 | -37 | 49 |  | 4,05 |
|  | R Inferior Parietal Lobe (IPL) | 40 | 57 | -37 | 49 |  | 3,82 |
|  |  |  |  |  |  |  |  |
| *Self > Control T3* |  |  |  |  |  |  |  |
|  | R Superior Medial Gyrus | 8 | 12 | 29 | 55 | 70 | 5,00 |
|  | R SMA | 6 | 15 | 11 | 64 |  | 4,13 |
|  | R SMA | 6 | 9 | 17 | 61 |  | 3,93 |
|  |  |  |  |  |  |  |  |
|  |  |  |  |  |  |  |  |
| *Reflected > Control T1* |  |  |  |  |  |  |  |
|  | L Lingual Gyrus | 18 | 3 | -79 | -2 | 55 | 5,44 |
|  |  |  |  |  |  |  |  |
| *Reflected > Control T3* |  |  |  |  |  |  |  |
|  | L Lingual Gyrus | 18 | 3 | -79 | -2 | 46 | 5,94 |
|  | R SMA | 6 | 12 | 17 | 61 | 67 | 4,85 |
|  | R Superior Frontal Gyrus | 6 | 15 | 26 | 55 |  | 4,16 |
|  |  |  |  |  |  |  |  |
| *Reflected Control*  *T3 > T1* | L Middle Cingulate Cortex (MCC) | 31 | -6 | -31 | 46 | 74 | 3,93 |
|  | L Posterior Cingulate Cortex (PCC) | 23 | -6 | -37 | 28 |  | 3,91 |
|  | L Middle Cingulate Cortex (MCC) | 31 | -6 | -37 | 52 |  | 3,62 |

Regions activated during the task based contrasts self > control and reflected > control at each time point separately (T1, T3) and for T3 > T1

*Note.* Names were based on the Automatic Anatomical Labeling (AAL) atlas.

**Table S.6**

Means, standard deviations, and correlations of all outcome variables

| *Variable* | *1* | *2* | *3* | *4* | *5* | *6* | *7* | *8* | *9* | *10* | *11* |
| --- | --- | --- | --- | --- | --- | --- | --- | --- | --- | --- | --- |
| 1.Satisfaction with choice |  |  |  |  |  |  |  |  |  |  |  |
| 2.Satisfaction with life | .27 |  |  |  |  |  |  |  |  |  |  |
| 3.Study commitment (U-MICS) | .79** | .45* |  |  |  |  |  |  |  |  |  |
| 4.Intrinsic motivation (SRQ) | .60** | .60** | .78** |  |  |  |  |  |  |  |  |
| 5.Identified regulation (SRQ) | .44* | .27 | .55** | .73** |  |  |  |  |  |  |  |
| 6.Academic adjustment (SACQ) | .54** | .24 | .63** | .62** | .46* |  |  |  |  |  |  |
| 7.Social adjustment (SACQ) | .31 | .14 | .53* | .55** | .48* | .44* |  |  |  |  |  |
| 8.Study dedication (UBES) | .78** | .20 | .83** | .68** | .56** | .49* | .26 |  |  |  |  |
| 9.Study absorption (UBES) | .75** | .37 | .72** | .59** | .41 | .63** | .29 | .73** |  |  |  |
| 10.Study vigor (UBES) | .72** | .26 | .85** | .72** | .51* | .71** | .47* | .77** | .82** |  |  |
| 11.Academic performance | .46* | .35 | .63** | .47* | .38 | .44* | .71** | .42 | .50* | .62** |  |
| *M* | 3.91 | 21.53 | 3.44 | 3.85 | 4.17 | 3.34 | 3.63 | 5.00 | 4.00 | 4.14 | 4.39 |
| *SD* | 1.03 | 5.72 | .73 | .60 | .45 | .73 | .82 | 1.44 | 1.43 | 1.38 | .78 |
| *Range* | 1 - 5 | 5 - 35 | 1 - 5 | 1 – 5 | 1 - 5 | 1 - 5 | 1 - 5 | 1 - 7 | 1 - 7 | 1 - 7 | 1 - 5 |

*Note:* **p*<.01. ***p*<.001
